# Supplementary material for: Development and application of simulation modelling for orthopaedic elective resource planning in England
Source: BMJ Open. 2023 Dec 22;13(12):e076221. doi: 10.1136/bmjopen-2023-076221 (PMC10748981; doi:10.1136/bmjopen-2023-076221)
Supplement: Supplementary data [file bmjopen-2023-076221supp002.pdf]

Supplementary Material 2
Table S1: OPCS-4 codes used to identify hip and knee arthroplasty surgery

PRIMARY TOTAL HIP REPLACEMENT		
4 Character Code	Description	
W37.1	Primary total prosthetic replacement of hip joint using cement	
W37.8	Other specified total prosthetic replacement of hip joint using cement	
W37.9	Unspecified total prosthetic replacement of hip joint using cement	
W38.1	Primary total prosthetic replacement of hip joint not using cement	
W38.8	Other specified total prosthetic replacement of hip joint not using cement	
W38.9	Unspecified total prosthetic replacement of hip joint not using cement	
W39.1	Primary total prosthetic replacement of hip joint NEC	
W39.8	Other specified other total prosthetic replacement of hip joint	
W39.9	Unspecified other total prosthetic replacement of hip joint	
W43.1	Primary total prosthetic replacement of other joint using cement NEC	
W43.8	Other specified total prosthetic replacement of other joint using cement NEC	
W43.9	Unspecified total prosthetic replacement of other joint using cement NEC	
W44.1	Primary total prosthetic replacement of other joint not using cement NEC	
W44.8	Other specified total prosthetic replacement of other joint not using cement NEC	
W44.9	Unspecified total prosthetic replacement of other joint not using cement NEC	
W45.1	Other primary total prosthetic replacement of other joint NEC	
W45.8	Other specified total prosthetic replacement of other joint NEC	
W45.9	Unspecified total prosthetic replacement of other joint NEC	
W52.1	Primary prosthetic replacement of articulation of bone using cement NEC	
W52.8	Other specified prosthetic replacement of articulation of bone using cement NEC	
W52.9	Unspecified prosthetic replacement of articulation of bone using cement NEC	
W53.1	Primary prosthetic replacement of articulation of bone not using cement NEC	
W53.8	Other specified prosthetic replacement of articulation of bone not using cement NEC	

W53.9	Unspecified prosthetic replacement of articulation of bone not using cement NEC	
W54.1	Primary prosthetic replacement of articulation of bone NEC	
W54.8	Other specified prosthetic replacement of articulation of bone NEC	
W54.9	Unspecified prosthetic replacement of articulation of bone NEC	
W93.1	Primary hybrid prosthetic replacement of hip joint using cemented acetabular component	
W93.8	Other specified hybrid prosthetic replacement of hip joint using cemented acetabular component	
W93.9	Unspecified hybrid prosthetic replacement of hip joint using cemented acetabular component	
W94.1	Primary hybrid prosthetic replacement of hip joint using cemented femoral component	
W94.8	Other specified hybrid prosthetic replacement of hip joint using cemented femoral component	
W94.9	Unspecified hybrid prosthetic replacement of hip joint using cemented femoral component	
W95.1	Primary hybrid prosthetic replacement of hip joint using cement NEC	
W95.8	Other specified hybrid prosthetic replacement of hip joint using cement	
W95.9	Unspecified hybrid prosthetic replacement of hip joint using cement	
**RESURFACING / RECONSTRUCTION**		
4 Character Code	Description	
W58.1	Primary resurfacing arthroplasty of joint	
W58.8	Other specified reconstruction of joint	
W58.9	Unspecified other reconstruction of joint	
**LOCATION OF SURGERY**		
4 Character Code	Description	
Z75.6	Acetabulum	
Z76.1	Head of femur	
Z84.3	Hip joint	
**REVISION HIP REPLACEMENT**		
4 Character Code	Description	

W37.0	Conversion from previous cemented total prosthetic replacement of hip joint	
W37.2	Conversion to total prosthetic replacement of hip joint using cement	
W37.3	Revision of total prosthetic replacement of hip joint using cement	
W37.4	Revision of one component of total prosthetic replacement of hip joint using cement	
W38.0	Conversion from previous uncemented total prosthetic replacement of hip joint	
W38.2	Conversion to total prosthetic replacement of hip joint not using cement	
W38.3	Revision of total prosthetic replacement of hip joint not using cement	
W38.4	Revision of one component of total prosthetic replacement of hip joint not using cement	
W39.0	Conversion from previous total prosthetic replacement of hip joint NEC	
W39.2	Conversion to total prosthetic replacement of hip joint NEC	
W39.3	Revision of total prosthetic replacement of hip joint NEC	
W39.5	Revision of one component of total prosthetic replacement of hip joint NEC	
W43.0	Conversion from previous cemented total prosthetic replacement of joint NEC	+ Site code
W43.2	Conversion to total prosthetic replacement of joint using cement NEC	+ Site code
W43.3	Revision of total prosthetic replacement of joint using cement NEC	+ Site code
W43.4	Revision of one component of total prosthetic replacement of joint using cement NEC	+ Site code
W44.0	Conversion from previous uncemented total prosthetic replacement of joint NEC	+ Site code
W44.2	Conversion to total prosthetic replacement of joint not using cement NEC	+ Site code
W44.3	Revision of total prosthetic replacement of joint not using cement NEC	+ Site code
W44.4	Revision of one component of total prosthetic replacement of joint not using cement NEC	+ Site code
W45.0	Conversion from previous total prosthetic replacement of joint NEC	+ Site code
W45.2	Conversion to total prosthetic replacement of joint NEC	+ Site code
W45.3	Revision of total prosthetic replacement of joint NEC	+ Site code
W45.5	Revision of one component of total prosthetic replacement of joint NEC	+ Site code
W46.2	Conversion to prosthetic replacement of head of femur using cement	

W46.3	Revision of prosthetic replacement of head of femur using cement	
W47.2	Conversion to prosthetic replacement of head of femur not using cement	
W47.3	Revision of prosthetic replacement of head of femur not using cement	
W48.2	Conversion to prosthetic replacement of head of femur NEC	
W48.3	Revision of prosthetic replacement of head of femur NEC	
W52.0	Conversion from previous cemented prosthetic replacement of articulation of bone NEC	+ Site code
W52.2	Conversion to prosthetic replacement of articulation of bone using cement NEC	+ Site code
W52.3	Revision of prosthetic replacement of articulation of bone using cement NEC	+ Site code
W53.0	Conversion from previous uncemented prosthetic replacement of articulation of bone NEC	+ Site code
W53.2	Conversion to prosthetic replacement of articulation of bone not using cement NEC	+ Site code
W53.3	Revision of prosthetic replacement of articulation of bone not using cement NEC	+ Site code
W54.0	Conversion from previous prosthetic replacement of articulation of bone NEC	+ Site code
W54.2	Conversion to prosthetic replacement of articulation of bone NEC	+ Site code
W54.3	Revision of prosthetic replacement of articulation of bone NEC	+ Site code
W57.4	Conversion to excision arthroplasty of joint	+ Site code
W58.0	Conversion from previous resurfacing arthroplasty of joint	+ Site code
W58.2	Revision of resurfacing arthroplasty of joint	+ Site code
W93.0	Conversion from previous hybrid prosthetic replacement of hip joint using cemented acetabular component	
W93.2	Conversion to hybrid prosthetic replacement of hip joint using cemented acetabular component	
W93.3	Revision of hybrid prosthetic replacement of hip joint using cemented acetabular component	
W94.0	Conversion from previous hybrid prosthetic replacement of hip joint using cemented femoral component	
W94.2	Conversion to hybrid prosthetic replacement of hip joint using cemented femoral component	
W94.3	Revision of hybrid prosthetic replacement of hip joint using cemented femoral component	
W95.0	Conversion from previous hybrid prosthetic replacement of hip joint using cement NEC	
W95.2	Conversion to hybrid prosthetic replacement of hip joint using cement NEC	
W95.3	Revision of hybrid prosthetic replacement of hip joint using cement NEC	

**PRIMARY TOTAL KNEE REPLACEMENT**		
4 Character Code	Description	
W40.1	Primary total prosthetic replacement of knee joint using cement	
W40.8	Other specified total prosthetic replacement of knee joint using cement	
W40.9	Unspecified total prosthetic replacement of knee joint using cement	
W41.1	Primary total prosthetic replacement of knee joint not using cement	
W41.8	Other specified total prosthetic replacement of knee joint not using cement	
W41.9	Unspecified total prosthetic replacement of knee joint not using cement	
W42.1	Primary total prosthetic replacement of knee joint NEC	
W42.8	Other specified other total prosthetic replacement of knee joint	
W42.9	Unspecified other total prosthetic replacement of knee joint	
O18.1	Primary hybrid prosthetic replacement of knee joint using cement	
O18.8	Other specified hybrid prosthetic replacement of knee joint using cement	
O18.9	Unspecified hybrid prosthetic replacement of knee joint using cement	
**PRIMARY UNICONDYLAR / UNICOMPARTMENTAL KNEE OPERATIONS**		
4 Character Code	Description	
W52.1	Primary prosthetic replacement of articulation of bone using cement NEC	Require combination with site + combination codes to ID
W52.8	Other specified prosthetic replacement of articulation of other bone using cement	Require combination with site + combination codes to ID

W52.9	Unspecified prosthetic replacement of articulation of other bone using cement	Require combination with site + combination codes to ID
W53.1	Primary prosthetic replacement of articulation of bone not using cement NEC	Require combination with site + combination codes to ID
W53.9	Unspecified prosthetic replacement of articulation of other bone not using cement	Require combination with site + combination codes to ID
W54.0	Conversion from previous prosthetic replacement of articulation of bone NEC	Require combination with site + combination codes to ID
W54.1	Primary prosthetic replacement of articulation of bone NEC	Require combination with site + combination codes to ID
W54.8	Other specified other prosthetic replacement of articulation of other bone	Require combination with site + combination codes to ID
W54.9	Unspecified other prosthetic replacement of articulation of other bone	Require combination with site + combination codes to ID
W58.1	Primary resurfacing arthroplasty of joint	Require combination with site + combination codes to ID
**SITE OF SURGERY**		
Z76.5	Lower end of femur NEC	
Z77.4	Upper end of tibia NEC	
Z78.7	Patella	Will need care in extracting as PFJ replacement will be coded with this
Z84.4	Patellofemoral joint	Will need care in extracting as PFJ replacement will be coded with this
Z84.5	Tibiofemoral joint	
Z84.6	Knee joint	
**REVISION KNEE REPLACEMENT**		
4 Character Code	Description	
W40.0	Conversion from previous cemented total prosthetic replacement of knee joint	

W40.2	Conversion to total prosthetic replacement of knee joint using cement	
W40.3	Revision of total prosthetic replacement of knee joint using cement	
W40.4	Revision of one component of total prosthetic replacement of knee joint using cement	
W41.0	Conversion from previous uncemented total prosthetic replacement of knee joint	
W41.2	Conversion to total prosthetic replacement of knee joint not using cement	
W41.3	Revision of total prosthetic replacement of knee joint not using cement	
W41.4	Revision of one component of total prosthetic replacement of knee joint not using cement	
W42.0	Conversion from previous total prosthetic replacement of knee joint NEC	
W42.2	Conversion to total prosthetic replacement of knee joint NEC	
W42.3	Revision of total prosthetic replacement of knee joint NEC	
W42.4	Attention to total prosthetic replacement of knee joint NEC	Plus Y03.2 (Renewal of prosthesis in organ NOC) or Y03.7 (Removal of prosthesis from organ NOC)
W42.5	Revision of one component of total prosthetic replacement of knee joint NEC	
W42.6	Arthrolysis of total prosthetic replacement of knee joint	
W58.0	Conversion from previous resurfacing arthroplasty of joint	
W58.2	Revision of resurfacing arthroplasty of joint	Require combination with site + combination codes to ID
O18.0	Conversion from previous hybrid prosthetic replacement of knee joint using cement	
O18.2	Conversion to hybrid prosthetic replacement of knee joint using cement	
O18.3	Revision of hybrid prosthetic replacement of knee joint using cement	
O18.4	Attention to hybrid prosthetic replacement of knee joint using cement	
W52.0	Conversion from previous cemented prosthetic replacement of articulation of bone NEC	Require combination with site + combination codes to ID
W52.2	Conversion to prosthetic replacement of articulation of bone using cement NEC	Require combination with site + combination codes to ID

W52.3	Revision of prosthetic replacement of articulation of bone using cement NEC	Require combination with site + combination codes to ID
W53.0	Conversion from previous uncemented prosthetic replacement of articulation of bone NEC	Require combination with site + combination codes to ID
W53.2	Conversion to prosthetic replacement of articulation of bone not using cement NEC	Require combination with site + combination codes to ID
W53.3	Revision of prosthetic replacement of articulation of bone not using cement NEC	Require combination with site + combination codes to ID
W54.0	Conversion from previous prosthetic replacement of articulation of bone NEC	Require combination with site + combination codes to ID
W54.2	Conversion to prosthetic replacement of articulation of bone NEC	Require combination with site + combination codes to ID
W54.3	Revision of prosthetic replacement of articulation of bone NEC	Require combination with site + combination codes to ID
W54.4	Attention to prosthetic replacement of articulation of bone NEC	Require combination with site + combination codes to ID
W55.3	Conversion to prosthetic interposition arthroplasty of joint	Require combination with site + combination codes to ID
W56.4	Conversion to interposition arthroplasty of joint NEC	Require combination with site + combination codes to ID
W57.4	Conversion to excision arthroplasty of joint	Require combination with site + combination codes to ID
W60.3	Conversion to arthrodesis and extra-articular bone graft NEC	Require combination with site + combination codes to ID
W61.3	Conversion to arthrodesis and articular bone graft NEC	Require combination with site + combination codes to ID
W64.1	Conversion to arthrodesis and internal fixation NEC	Require combination with site + combination codes to ID
W64.2	Conversion to arthrodesis and external fixation NEC	Require combination with site + combination codes to ID
