# Supplementary material for: Development and application of simulation modelling for orthopaedic elective resource planning in England
Source: BMJ Open. 2023 Dec 22;13(12):e076221. doi: 10.1136/bmjopen-2023-076221 (PMC10748981; doi:10.1136/bmjopen-2023-076221)
Supplement: Author's manuscript [file bmjopen-2023-076221.draft_revisions.pdf]

BMJ Open

BMJ Open is committed to open peer review. As part of this commitment we make the peer review history of every article we publish publicly available.

When an article is published we post the peer reviewers' comments and the authors' responses online. We also post the versions of the paper that were used during peer review. These are the versions that the peer review comments apply to.

The versions of the paper that follow are the versions that were submitted during the peer review process. They are not the versions of record or the final published versions. They should not be cited or distributed as the published version of this manuscript.

BMJ Open is an open access journal and the full, final, typeset and author-corrected version of record of the manuscript is available on our site with no access controls, subscription charges or pay-per-view fees (http://bmjopen.bmj.com).

If you have any questions on BMJ Open's open peer review process please email info.bmjopen@bmj.com

BMJ Open

Post-COVID orthopaedic elective resource planning using simulation modelling

Journal:	*BMJ Open*
Manuscript ID	bmjopen-2023-076221
Article Type:	Original research
Date Submitted by the Author:	31-May-2023
Complete List of Authors:	Harper, Alison; University of Exeter Medical School, NIHR PenARC<br>Monks, Thomas; University of Exeter Medical School, NIHR PenARC<br>Wilson, Rebecca; National Institute for Health, Population Health Sciences<br>Redaniel, Maria Theresa; National Institute for Health, Population Health Sciences; University of Bristol, Population Health Sciences<br>Eyles, Emily; National Institute for Health, Population Health Sciences; University of Bristol, Population Health Sciences<br>Jones, Tim; National Institute for Health, Population Health Sciences; University of Bristol, Population Health Sciences<br>Penfold, Chris; University of Bristol, Musculoskeletal Research Unit<br>Elliott, Andrew; Southmead Hospital<br>Keen, Tim; North Bristol NHS Foundation Trust<br>Pitt, Martin; University of Exeter, University of Exeter: Medical School<br>Blom, Ashley; University of Sheffield<br>Whitehouse, Michael; North Bristol NHS Foundation Trust<br>Judge, Andrew; University of Bristol, Musculoskeletal Research Unit
Keywords:	ORTHOPAEDIC & TRAUMA SURGERY, Information management < BIOTECHNOLOGY & BIOINFORMATICS, World Wide Web technology < BIOTECHNOLOGY & BIOINFORMATICS, Organisation of health services < HEALTH SERVICES ADMINISTRATION & MANAGEMENT

SCHOLARONE™
Manuscripts

BMJ

I, the Submitting Author has the right to grant and does grant on behalf of all authors of the Work (as defined in the below author licence), an exclusive licence and/or a non-exclusive licence for contributions from authors who are: i) UK Crown employees; ii) where BMJ has agreed a CC-BY licence shall apply, and/or iii) in accordance with the terms applicable for US Federal Government officers or employees acting as part of their official duties; on a worldwide, perpetual, irrevocable, royalty-free basis to BMJ Publishing Group Ltd ("BMJ") its licensees and where the relevant Journal is co-owned by BMJ to the co-owners of the Journal, to publish the Work in this journal and any other BMJ products and to exploit all rights, as set out in our licence.

The Submitting Author accepts and understands that any supply made under these terms is made by BMJ to the Submitting Author unless you are acting as an employee on behalf of your employer or a postgraduate student of an affiliated institution which is paying any applicable article publishing charge ("APC") for Open Access articles. Where the Submitting Author wishes to make the Work available on an Open Access basis (and intends to pay the relevant APC), the terms of reuse of such Open Access shall be governed by a Creative Commons licence – details of these licences and which Creative Commons licence will apply to this Work are set out in our licence referred to above.

Other than as permitted in any relevant BMJ Author's Self Archiving Policies, I confirm this Work has not been accepted for publication elsewhere, is not being considered for publication elsewhere and does not duplicate material already published. I confirm all authors consent to publication of this Work and authorise the granting of this licence.

For peer review only

POST-COVID ORTHOPAEDIC ELECTIVE RESOURCE PLANNING USING SIMULATION MODELLING

Alison Harper[1], Thomas Monks[1], Rebecca Wilson[2], Maria Theresa Redaniel[2], Emily Eyles[2], Tim Jones[2], Chris Penfold[3], Andrew Elliott[4], Tim Keen[4], Martin Pitt[1], Ashley Blom[5], Michael Whitehouse[4], and Andrew Judge[3]

[1]The National Institute for Health and Care Research Applied Research Collaboration West (NIHR PenARC) at University of Exeter a.l.harper@exeter.ac.uk t.m.w.monks@exeter.ac.uk m.pitt@exeter.ac.uk

[2] The National Institute for Health and Care Research Applied Research Collaboration West (NIHR ARC West) at University Hospitals Bristol and Weston NHS Foundation Trust, UK; Population Health Sciences, Bristol Medical School, University of Bristol, UK Rebecca.wilson@bristol.ac.uk Theresa.redaniel@bristol.ac.uk Emily.eyles@bristol.ac.uk timothy.jones@bristol.ac.uk

[3]NIHR Biomedical Research Centres University Hospitals Bristol and Weston NHS FT and University of Bristol Andrew.judge@bristol.ac.uk chris.penfold@bristol.ac.uk

[4]North Bristol NHS Foundation Trust tim.keen@nbt.nhs.uk Andrew.elliott@nbt.nhs.uk Michael.Whitehouse@bristol.ac.uk

[5]Faculty of Health, University of Sheffield a.blom@sheffield.ac.uk

Correspondence to Alison Harper: a.l.harper@exeter.ac.uk

Keywords: Orthopedics, Benchmarking, Mobile Applications, Simulation, Elective

Word count: 3274

ABSTRACT

Objectives: To develop a simulation model to support orthopaedic elective capacity planning.

Methods: An open-source, generalisable discrete-event simulation was developed, including a web-based application. The model used anonymised patient records between 2016-2019 of elective orthopaedic procedures from an NHS Trust in England. In this paper, it is used to investigate scenarios including resourcing (beds and theatres) and productivity (lengths-of-stay, delayed discharges, theatre activity) to support planning for meeting new NHS targets aimed at reducing elective orthopaedic surgical backlogs in a proposed ring fenced orthopaedic surgical facility. The simulation is interactive and intended for use by health service planners and clinicians.

Results: A higher number of beds (65-70) than the proposed number (40 beds) will be required if lengths-of-stay and delayed discharge rates remain unchanged. Reducing lengths-of-stay in line with national benchmarks reduces bed utilisation to an estimated 60%, allowing for additional theatre activity such as weekend working. Further, reducing the proportion of patients with a delayed discharge by 75% reduces bed utilisation to below 40%, even with weekend working. A range of other scenarios can also be investigated directly by NHS planners using the interactive web app.

Conclusions: The simulation model is intended to support capacity planning of orthopaedic elective services by identifying a balance of capacity across theatres and beds and predicting the impact of productivity measures on capacity requirements. It is applicable beyond the study site and can be adapted for other specialties.

Strengths and Limitations of this study:

For peer review only

- The simulation model provides rapid quantitative estimates to support post-COVID elective services recovery toward medium-term elective targets.
- Parameter combinations include changes to both resourcing and productivity.
- The interactive web app enables intuitive parameter changes by users while underlying source code can be adapted or re-used for similar applications.
- Patient attributes such as complexity are not included in the model but are reflected in variables such as length-of-stay and delayed discharge rates.
- Theatre schedules are simplified, incorporating the five key orthopaedic elective surgical procedures.

INTRODUCTION

Elective joint replacement comprises one of the highest volumes of elective procedures worldwide [1]. In the UK, orthopaedics has been the specialty under most pressure in terms of performance against National Health Service (NHS) elective operational standards [2]. Prior to the COVID-19 pandemic, increased waiting times for elective orthopaedic surgery reflected limited NHS resources and the competing demands of rising emergency admissions that affect mixed sites. This has been particularly problematic during the winter months when emergency demand for hospital care is high [3].

Hip and knee replacement have been shown to be strongly cost effective from both a societal and a health system perspective compared to non-surgical treatment [4]. Procedures that are not delayed are more cost effective than delayed intervention, while patients delayed for surgery for more than 180 days have been shown to be at higher risk of poor outcomes [4-6]. Additionally, reviews have found that low surgical volume is associated with longer procedure times and lengths-of-stay, and poorer patient outcomes including risk of revision [7-9]. Given substantial evidence that surgeons undertaking low volumes of specific surgical activities may increase costs and result in less favourable outcomes for patients, Getting it Right First Time (GIRFT) [10] published a set of recommendations, such as ring-fencing beds and improving criteria-led discharge, aimed at reducing the significant variation found in practice. While successful [11], performance against core national standards have continued to deteriorate, attributable to increasing demand and lack of available capacity [2, 12].

The effect of the COVID-19 pandemic on elective orthopaedic services has been to compound ongoing challenges, with larger waiting lists and a steep decline in performance. Following the pandemic, a deterioration has been found in the health of patients who have had elective joint replacement postponed [14]. In response to this situation, the government has committed two-year revenue allocations to support Integrated Care Boards (ICBs) to expand capacity. ICBs and primary and secondary care providers are required to develop plans to meet national objectives and local priorities, in particular to eliminate elective waits of over 65 weeks by March 2024 [15]. While central capital funding will be key to achieving this, maximising use of resources and reducing lengths-of-stay and delayed discharges are required to make effective use of available capacity, and are associated with improvements in patient care [12].

ICBs are therefore currently working to secure capital funds by delivering business cases that evidence optimal capacity and productivity configurations considering activity, workforce, capital requirements and potential revenue. Simulation modelling can be used as a planning tool to provide supporting evidence by modelling various configurations of bed numbers, theatre capacity, and ward stays to estimate resultant surgical throughput. In this paper, we describe the development and implementation of an interactive, free and open-source simulation model to support planning of ring-fenced elective orthopaedic capacity. The model is co-designed with North Bristol NHS Trust (NBT), and is designed to be reusable, generalisable and to provide rapid information for clinicians, business and service managers across a range of scenarios relevant to new orthopaedic capacity planning.

For peer review only

METHODS

We developed a discrete-event simulation (DES) model (programmed in Python 3.8) of surgical activity and ward stay in a proposed ring-fenced orthopaedic facility. DES allows processes and pathways to be modelled at individual patient level, and to explore the potential impact of changes to the system without the costs and risks associated with real-world changes. It has been used for patient flow management, resource allocation, and scheduling, for example in sexual health [15], stroke pathways [16], and orthopaedics [17-18]. The DES was developed to have the flexibility to answer a range of 'what-if' questions of interest to NBT, and is generalisable to other NHS Trusts for orthopaedic elective planning.

The DES model is free and open source, and is available as a web app: https://hospital-efficiency-project.streamlit.app/. To preserve code, we have permanently archived it using Zenodo (HEP | Zenodo). All code has an MIT license allowing free reuse and adaptation by researchers, industry and the NHS. Our app provides a user-friendly, interactive interface for the DES, including instructions for use and documentation, allowing NHS staff to experiment with model parameters and generate immediate results without the need to download and install software. The model can also be adapted to other specialties.

The model is documented using STRESS reporting guidelines [20], available in supplementary material 1.

Data and Setting

NBT serves a population of approximately 1-million people, with an age-profile in line with England. Routinely collected data from the NHS Trust was used to identify patients receiving elective joint replacement between January 2016 and December 2019.

The Trust's electronic health records (EHR) were used to identify elective joint replacements using a combination of OPCS4 procedure and surgical site codes (Supplementary material 2). Five core elective orthopedic surgical procedure types were identified and verified. A small number of short day-case 'hip resurfacing' surgeries [n=52] were removed from the dataset as they rarely utilize bed capacity. The five remaining surgical types were classified into two classes: (I) *Primary [87%]:* (primary hip replacement [p-THR n=3057; 51%], primary knee replacement [p-TKR; n=2302; 38%], uni-compartmental knee replacement [p-UKR; n=679; 11%]); (II) *Revision [13%]:* (revision hip replacement [r-THR; n=482; 55%], revision knee replacement [r-TKR; n=392; 45%]). Most patients did not remain in hospital once they were medically fit for discharge, however a proportion of patients in the EHR had a recorded *medically fit for discharge* date which preceded their actual discharge date (n=529; 7.6%).

The DES requires parameters describing patient lengths-of-stay, hence statistical probability distributions were fitted to each category of surgical procedure using the EHR data. The length-of-stay parameters [procedure, mean days (u), standard deviation days (sd)] are: p-THR, u=4.4, sd=2.9; p-TKR, u=4.7, sd=2.8; p-UKR, u=2.9, sd=2.1; r-THR, u=6.9, sd=7.0; r-TKR, u=7.2, sd=7.6; delayed discharge, u=16.5; sd=15.1), which are converted to lognormal parameters within the model. The mean lengths-of-stay are high against national benchmarks, and a key focus of future activity is to reduce lengths-of-stay [12, 23].

Orthopaedic surgical pathway

The DES model is a simplified, high-level representation of the system of interest, which simulates individual patient flow through the system over time. Our model assumes an infinite waiting list. Baseline surgical theatre scheduling rules define how patients enter the simulation model according to their surgical class (Primary or Revision). Baseline rules are:

- Three theatre sessions per day, five days per week with no weekend activity.
- Morning and afternoon sessions schedule either: one Revision *or* two Primary surgeries.
- Evening session schedules one Primary surgery.

For peer review only

Baseline resources for the model are 40 beds and 4 theatres. The DES samples a length-of-stay for each simulated patient from a lognormal distribution with parameters calculated from the previously fitted distributions by procedure type. A length-of-stay is sampled for delayed patients. The simulation can be run using baseline parameters, and additional scenarios can be run by changing these parameters to determine the effects on model outputs, primarily surgical throughput. Figure 1 shows the organisation of surgical activity and ward stay.

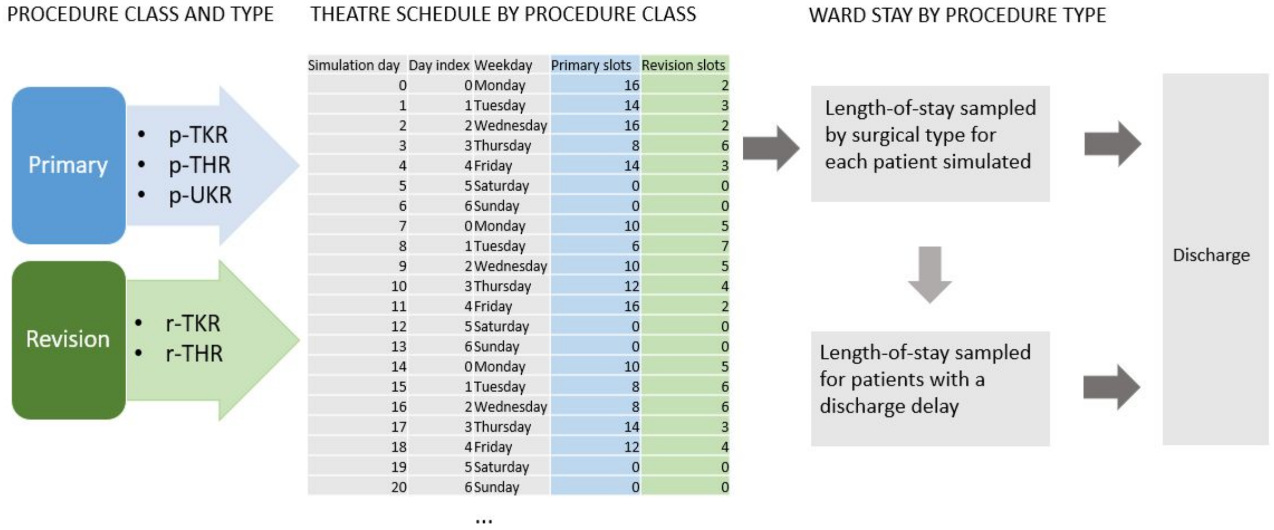

Figure 1: Conceptual organisation of ring-fenced orthopaedic activity and ward stay

Model objectives

With the overall objective of maximising surgical throughput, the effects of changing model inputs can be investigated alone or in combination as experimental scenarios. These include:

- The number of theatres;
- The number of ring-fenced beds;
- Patient lengths-of-stay, including delayed discharges;
- Proportion of patients with a delayed discharge;
- Effects of running evening or weekend theatre sessions;
- Changes to surgical scheduling, for example scheduling revision surgery (with longer, more variable lengths-of-stay) earlier in the week.

These questions are all in line with GIRFT priorities, which include accepting day surgery as default (where a bed is available as back-up), improving theatre utilisation and best practice care, and focused enhanced recovery. Higher surgical volumes, dedicated theatre teams, and enhanced post-operative recovery is expected to improve patient outcomes. In turn, it is expected that lengths-of-stay will reduce, and standardised clinical pathways and discharge planning are likely to reduce discharge delays [12, 20].

The model outputs:

- *Total surgical throughput*. The primary goal of central capital funding allocations is to efficiently maximize surgical throughput [14], so the configuration which best achieves this - within other constraints relevant to service planners such as workforce availability [21] - is a key output.
- *Bed utilisation per day of week*. For each experimental scenario, mean bed utilisation (occupancy) is outputted daily over model runtime. While there is no ideal average bed utilisation figure (which is dependent upon many factors), it is commonly accepted that mean occupancies greater than 85-90%

For peer review only

can expect regular bed crises [22]. The results of excessive bed utilisation in the model can be seen as 'lost slots', where no bed is available for a patient scheduled for surgery. An additional consideration is that GIRFT recommend the extension of therapy services to support patient mobility goals toward discharge on any day with elective operating [20]. Therefore, theatre scheduling decisions (e.g., 5-7 day service) are dependent upon the availability of weekend staff.

- *Lost theatre slots for system reasons*. While beds are protected from outlying emergency admissions in a ring-fenced scenario, the balance of beds to theatre activity is a critical question. In the model, where patients are scheduled to arrive for surgery but no bed is available, the theatre slot is lost. In reality, other system behaviours will account for some of these lost slots. For example, the slot may be lost for patient reasons such as illness (i.e. the patient doesn't attend for surgery or is deemed not fit for surgery at the point of admission); bed management activities may free up beds; or patients may be transferred to the acute hospital. In the model, lost slots (per day of week) are an indication of a mismatch between demand (theatre scheduling) and capacity (bed utilisation). In NBT, an average of 4.75 slots are lost per week for patient reasons, and a further 2.5 for system reasons.

Patient and public involvement

Patients and the public were consulted in a workshop for suggestions and comments to inform the development of the grant that supported this work, and a further workshop informed scenarios used in model development.

Role of the funding source

The funders had no role in any of the following: the study design; the collection, analysis, and interpretation of data; the writing of the report; the decision to submit the paper for publication.

RESULTS

A set of 72 indicative experimental parameter configurations were investigated (summarised in Table 1) and described here:

Bed numbers between 30 and 70 beds (in increments of 5 beds; a total of 9 bed parameters) were investigated with each of two theatre schedules. The Baseline theatre schedule is as described in the Methods section, with 5-day working. The Baseline + weekend schedule uses the same daily theatre allocations and theatre numbers, with 7-day working. For each of these parameter changes, four scenarios were run for lengths-of-stay (baseline, 25% baseline) and for proportion delayed (baseline, 25% baseline). This totals 72 combinations.

Table 1: Summary of scenarios varying procedure lengths-of-stay (los), bed numbers, proportion of patients with a delayed discharge (prop), and daily theatre schedule.

**Scenario**	**Length-of-stay (los): 2 parameters**	**Proportion delayed (prop): 2 parameters**	**Beds: 9 parameters**	**Schedule: 2 parameters**
Scenario 1	Baseline	Baseline	30 – 70 (in intervals of 5)	Baseline<br>Baseline + weekend
Scenario 2	0.25x Baseline	0.25x Baseline	30 – 70 (in intervals of 5)	Baseline<br>Baseline + weekend
Scenario 3	Baseline	0.25x Baseline	30 – 70 (in intervals of 5)	Baseline<br>Baseline + weekend
Scenario 4	0.25x Baseline	Baseline	30 – 70 (in intervals of 5)	Baseline<br>Baseline + weekend

For peer review only

The baseline procedure lengths-of-stay and proportion of patients with a delayed discharge are historical values described in the Methods section. As a goal of capacity planning is to reduce lengths-of-stay and delayed discharge, these are considered maximum values (high_los, prop_high), with minimum values set at 25% of baseline (low_los, prop_low). In all cases, the length-of-stay parameters for those patients with a discharge delay remain at the baseline value.

The results are plotted in Figure 2. The top row displays the mean total daily surgical throughput for each scenario, and for each theatre schedule. The middle row is mean daily bed utilisation. The bottom row displays 'lost slots', estimating the extent of the mismatch between patients scheduled and beds available.

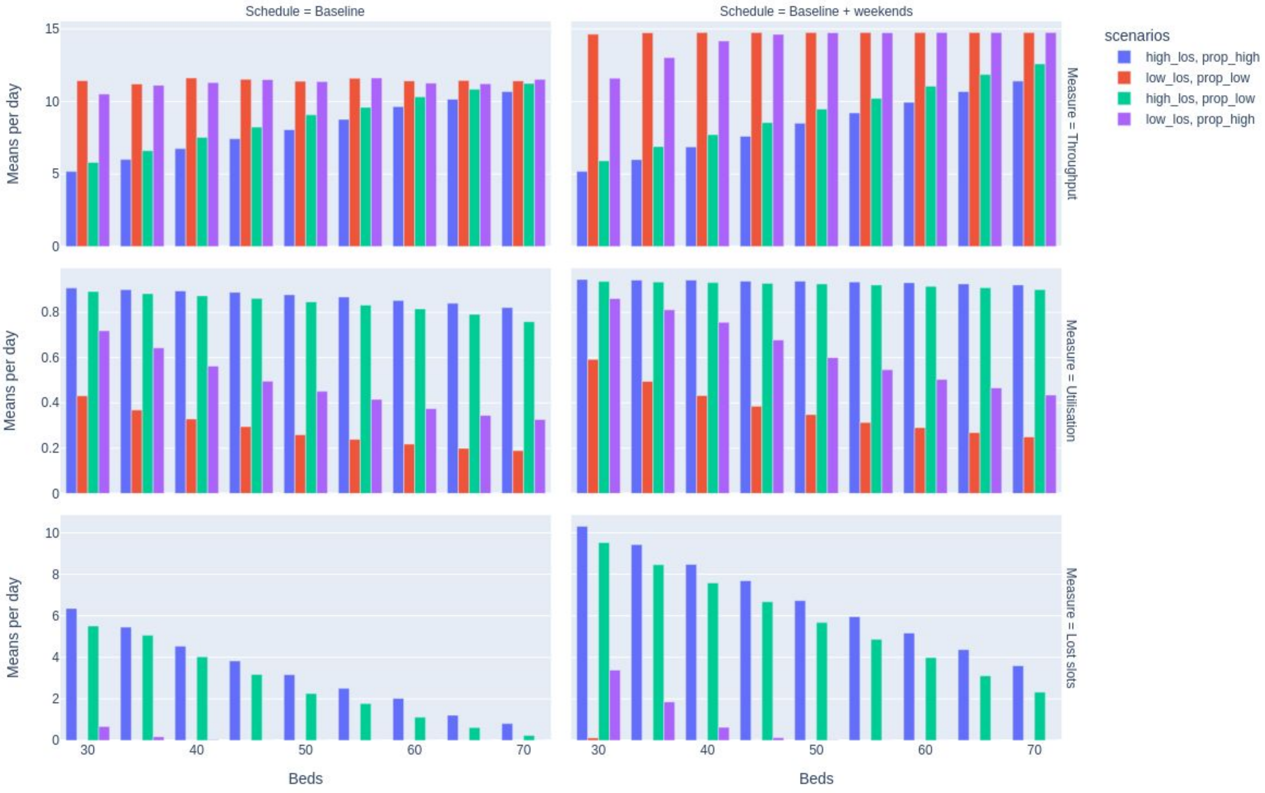

Figure 2: Results of simulations across 30-70 beds, for each of 2 theatre schedules with 4 combinations of lengths-of-stay (baseline:high_los; baseline x0.25: low_los) and proportion delayed (baseline:prop_high; baseline x0.25: prop_los) for mean daily total surgical throughput, bed utilisation, and lost slots.

The results show that the system is more sensitive to changes in procedure lengths-of-stay than to changes in the proportion of patients delayed, despite the long mean lengths-of-stay for delayed patients. At current procedure lengths-of-stay (high_los), bed utilisation is high with both current and reduced delayed discharges (Scenarios 1 and 3). With reduced lengths-of-stay, the effects of reducing delayed discharges on required bed numbers is more significant, substantially reducing bed utilisation.

In the case of no weekend activity, a higher number of beds (65-70) than the proposed value (40 beds) will be required if lengths-of-stay remain unchanged. At this level of bed utilisation, reducing delayed discharges has little impact on required bed numbers. However, reducing lengths-of-stay in line with national benchmarks has enough impact on bed utilisation to allow for additional theatre activity.

Where lengths-of-stay can be reduced, weekend operating theatre activity (remaining at 4 theatres) increases surgical throughput, and beds remain underutilised in all cases above 40 beds. Reducing the proportion of patients with delayed discharge further reduces bed requirements to approximately 30 beds. Where lengths-of-stay remain at baseline values, weekend theatre activity cannot be considered, as bed utilisation and resultant

For peer review only - http://bmjopen.bmj.com/site/about/guidelines.xhtml

lost slots are unacceptably high, even up to 70 beds. Users can investigate scenarios between these extreme values to gain realistic expectations of required bed and theatre numbers.

DISCUSSION

Our generalisable, open access application allows those involved in planning the development and utilisation of ring-fenced elective orthopaedic units to rapidly model different scenarios to predict delivery of elective surgical care. The model is adaptable to use local data for other units to model likely scenarios when planning activity. With minimal adaptation, it can also be applied to the delivery of other types of elective surgery.

Our experimental scenarios found that reducing lengths-of-stay in line with national benchmarks can increase surgical throughput and allow for additional theatre activity given the bed and theatre numbers initially proposed. We investigated weekend working, but the model also allows estimates to be obtained for increased theatre numbers, increased theatre utilisation (more procedures per day), and changes to theatre activity (e.g., scheduling more complex surgery early in the week). However, if lengths-of-stay remain unchanged, proposed bed numbers in this instance will be inadequate. As procedure lengths-of-stay reduce, the effects of reducing the number of patients with a delayed discharge also become more significant. Reducing the length of the delay will similarly reduce bed utilisation and increase throughput, and the results of this can also be investigated using the simulation model.

A strength of simulation modelling is the use of underlying stochastic distributions using real-world data, as planning by average occupancy will not provide adequate reserve capacity to manage the variation seen in patient lengths-of-stay [25]. Our open approach to modeling is a further strength, as a range of scenario combinations can be investigated by users to support planning using the web app. Additionally, the model is available for re-use either through re-parameterisation or adaptation. There are limitations to the use of simulation modeling. Assumptions and simplifications are required to convert a real-world problem into a computer representation. We assume that all historical (pre-pandemic) lengths-of-stay distributions fit current lengths-of-stay for baseline modelling. We do not account for patient frailty or other patient attributes beyond length-of-stay and the proportion of delayed discharges, and it is possible that patients who have been delayed are more complex. Additionally, our theatre scheduling rules include only the five main elective orthopaedic procedures. While other procedures may utilize theatre activity, they are not accounted for in bed planning, although simpler procedures will more likely to be performed as day cases, and more complex procedures (such as spinal surgery) will be performed in a main hospital setting with high dependency facilities.

Previous modelling and simulation work has focused on resource sharing for elective and non-elective joint replacement [17] and detailed studies of individual orthopaedic services [18, 23]. Although the need for reusable models for orthopaedic wait list planning is recognised [24], no free and open-source models to support resourcing and efficiency of elective services have been found. Our model provides quantitative outputs estimating the effects on surgical throughput (per procedure); daily bed utilisation of changes to bed and theatre capacity, theatre scheduling, patient lengths-of-stay and discharge delays; and lost theatre slots, representing system pressure on beds. The model is intended for use by health services planners and clinicians, and is available as a free web-app to address usability and accessibility of results. It is being used to evidence service configurations for the business case in NBT, and is more widely applicable.

CONCLUSIONS

Post-pandemic, pressure to restore elective surgeries against new interim national targets necessitates efficient and effective use of allocated public funds. This planning is happening rapidly, and on a large scale across the UK. Simulation modelling offers an effective method for planning elective services, identifying a balance of capacity across theatres and beds, and predicting the impact of productivity measures on capacity requirements. The model developed in this study is being used to provide quantitative support for accessing central capital funds, enabling discussion and evidence for the most efficient use of new resourcing. The model has been

For peer review only

developed to offer a transferable solution for supporting both orthopaedic elective recovery, and with minor adaptations, recovery of other elective services.

Data availability statement

Data may be obtained from a third party and are not publicly available. The data used in the study are collected by the North Bristol NHS Trust (NBT) as part of their care and support. Sharing of anonymised data with the University of Bristol was underpinned by a data sharing agreement and solely covers the purposes of this study. Data requests can be made through the NBT.

Code, input parameters and output data used for results in this paper are available on GitHub: AliHarp/HEP: Model of orthopaedic ward (github.com) and are permanently archived using Zenodo (HEP | Zenodo). All are licensed using MIT permissive license MIT License | Choose a License.

Patient consent for publication

Not applicable.

Ethics approval

We were provided with pseudonymised North Bristol Trust hospital admissions data under the NIHR ARC West Partnership Agreement. The project received ethical approval from the University of Bristol Faculty of Health Sciences ethical review board on 3 November 2020 (ref# 109024).

Funding

This study was funded by the Health Data Research (HDR) UK South West Better Care Partnership (#6.12). AH, TM and MP are supported by the National Institute for Health Research Applied Research Collaboration South West Peninsula. RW, EE, TJ and MTR's time was supported by the National Institute for Health Research Applied Research Collaboration West (NIHR ARC West). AJ, MW, AB and CP were supported by the NIHR Biomedical Research Centre at University Hospitals Bristol and Weston NHS Foundation Trust and the University of Bristol. The views expressed in this publication are those of the author(s) and not necessarily those of the National Institute for Health Research or the Department of Health and Social Care.

Competing interests None declared

Contributors AH drafted the original manuscript. AH, TM, and MP developed the model. All authors contributed to the conception and planning of this project and all reviewed and revised the manuscript. MTR and AJ supervised the project. MTR is the guarantor for the project.

REFERENCES

[1] Meara, J.G., Leather, A.J., Hagander, L., Alkire, B.C., Alonso, N., Ameh, E.A., Bickler, S.W., Conteh, L., Dare, A.J., Davies, J. and Mérisier, E.D., 2015. Global Surgery 2030: evidence and solutions for achieving health, welfare, and economic development. *The lancet*, *386*(9993), pp.569-624.

[2] The Kings Fund. 2021. Waiting times for elective (non-urgent) treatment: referral to treatment (RTT) Available at: Waiting times for elective (non-urgent) treatment: referral to treatment (RTT) | The King's Fund (kingsfund.org.uk)

[3] Alderson, D., 2018. Winter pressures that last all year. *The Bulletin of the Royal College of Surgeons of England*, *100*(2), pp.53-53.

[4] Lan, R.H., Yu, J., Samuel, L.T., Pappas, M.A., Brooks, P.J. and Kamath, A.F., 2020. How are we measuring cost-effectiveness in total joint arthroplasty studies? Systematic review of the literature. *The Journal of arthroplasty*, *35*(11), pp.3364-3374.

For peer review only

[5] Kapstad, H., Rustøen, T., Hanestad, B.R., Moum, T., Langeland, N. and Stavem, K., 2007. Changes in pain, stiffness and physical function in patients with osteoarthritis waiting for hip or knee joint replacement surgery. *Osteoarthritis and cartilage*, *15*(7), pp.837-843.

[6] Scott, C.H., MacDonald, D.J. and Howie, C.R., 2019. 'Worse than death'and waiting for a joint arthroplasty. *The bone & joint journal*, *101*(8), pp.941-950.

[7] Lau, R.L., Perruccio, A.V., Gandhi, R. and Mahomed, N.N., 2012. The role of surgeon volume on patient outcome in total knee arthroplasty: a systematic review of the literature. *BMC musculoskeletal disorders*, *13*(1), pp.1-10.

[8] Pamilo, K.J., Peltola, M., Mäkelä, K., Häkkinen, U., Paloneva, J. and Remes, V., 2013. Is hospital volume associated with length-of-stay, re-admissions and reoperations for total hip replacement? A population-based register analysis of 78 hospitals and 54,505 replacements. *Archives of orthopaedic and trauma surgery*, *133*, pp.1747-1755.

[9] Sayers, A., Steele, F., Whitehouse, M.R., Price, A., Ben-Shlomo, Y. and Blom, A.W., 2020. Association between surgical volume and failure of primary total hip replacement in England and Wales: findings from a prospective national joint replacement register. *BMJ open*, *10*(9), p.e033045.

[10] GIRFT. 2015. A national review of adult elective orthopaedic services in England GETTING IT RIGHT FIRST TIME. Available at: Layout 1 (gettingitrightfirsttime.co.uk)

[11] GIRFT. 2020. Getting it Right in Orthopaedics: A follow-up on the GIRFT national specialty report on orthopaedics. Available at: Layout 1 (gettingitrightfirsttime.co.uk)

[12] Wall, J., Ray, S. and Briggs, T.W., 2022. Delivery of elective care in the future. *Future healthcare journal*, *9*(2), p.144.

[13] Pietrzak, J.R.T., Maharaj, Z., Erasmus, M., Sikhauli, N., Cakic, J.N. and Mokete, L., 2021. Pain and function deteriorate in patients awaiting total joint arthroplasty that has been postponed due to the COVID-19 pandemic. *World Journal of Orthopedics*, *12*(3), p.152.

[14] NHS. 2022. Delivery Plan for Tackling the Covid-19 backlog of elective care. Available at: https://www.england.nhs.uk/coronavirus/wp-content/uploads/sites/52/2022/02/C1466-delivery-plan-for-tackling-the-covid-19-backlog-of-elective-care.pdf

[15] Mohiuddin, S., Gardiner, R., Crofts, M., Muir, P., Steer, J., Turner, J., Wheeler, H., Hollingworth, W. and Horner, P.J., 2020. Modelling patient flows and resource use within a sexual health clinic through discrete event simulation to inform service redesign. *BMJ open*, *10*(7), p.e037084.

[16] Allen, M., Pearn, K., Monks, T., Bray, B.D., Everson, R., Salmon, A., James, M. and Stein, K., 2019. Can clinical audits be enhanced by pathway simulation and machine learning? An example from the acute stroke pathway. *BMJ open*, *9*(9), p.e028296.

[17] Persson, M., Hvitfeldt-Forsberg, H., Unbeck, M., Sköldenberg, O.G., Stark, A., Kelly-Pettersson, P. and Mazzocato, P., 2017. Operational strategies to manage non-elective orthopaedic surgical flows: a simulation modelling study. *BMJ open*, *7*(4), p.e013303.

[18] Anderson, G.H., Jenkins, P.J., McDonald, D.A., Van Der Meer, R., Morton, A., Nugent, M. and Rymaszewski, L.A., 2017. Cost comparison of orthopaedic fracture pathways using discrete event simulation in a Glasgow hospital. *BMJ open*, *7*(9), p.e014509.

[19] Monks, T., Currie, C.S., Onggo, B.S., Robinson, S., Kunc, M. and Taylor, S.J., 2019. Strengthening the reporting of empirical simulation studies: Introducing the STRESS guidelines. *Journal of Simulation*, *13*(1), pp.55-67.

For peer review only

[20] GIRFT. 2023. Orthopaedic Elective Surgery Guide to delivering perioperative ambulatory care for patients with hip and knee pain requiring joint replacement surgery. Available at: SWAOC-Hip-and-Knee-Delivery-Guide-March-2023-FINAL-V1-1.pdf (gettingitrightfirsttime.co.uk)

[21] Mahase, E., 2022. Health leaders question absence of workforce strategy in NHS elective care recovery plan.

[22] Bain, C.A., Taylor, P.G., McDonnell, G. and Georgiou, A., 2010. Myths of ideal hospital occupancy. *Medical Journal of Australia*, *192*(1), pp.42-43.

[23] Baril, C., Gascon, V. and Cartier, S., 2014. Design and analysis of an outpatient orthopaedic clinic performance with discrete event simulation and design of experiments. *Computers & Industrial Engineering*, *78*, pp.285-298.

[24] Boyle, L. and Mackay, M., 2022, December. A Reusable Discrete Event Simulation Model for Improving Orthopedic Waiting Lists. In *2022 Winter Simulation Conference (WSC)* (pp. 973-984). IEEE.

[25] Monks, T., Worthington, D., Allen, M., Pitt, M., Stein, K. and James, M.A., 2016. A modelling tool for capacity planning in acute and community stroke services. *BMC health services research*, *16*(1), pp.1-8.

For peer review only

Supplementary Material 1
STRESS-DES: Strengthening the Reporting of Empirical Simulation Studies
A standardised checklist to improve the reporting of discrete-event simulation models

Monks, T., Currie, C. S., Onggo, B. S., Robinson, S., Kunc, M., & Taylor, S. J. (2019). Strengthening the reporting of empirical simulation studies: Introducing the STRESS guidelines. Journal of Simulation, 13(1), 55-67. https://doi.org/10.1080/17477778.2018.1442155

1.0 Objectives
1.1 Purpose of the model

The HEP orthopaedic capacity planning model provides a tool for healthcare planners, managers, and clinicians to support capacity and efficiency decisions for orthopaedic services. It includes planning for two classes of surgery:

- Primary
- Revision

With five surgical types:

- Primary total hip replacement
- Primary total knee replacement
- Primary unicompartmental knee replacement
- Revision total hip replacement
- Revision total knee replacement

1.2 Model outputs

For each scenario configuration, the model outputs:

(i) Bed utilisation (mean/CI per day/weekday)

(ii) Total mean surgical throughput (by surgical type).

(iii) 'Lost theatre slots' which represent a mismatch between demand (patients scheduled for surgery), and capacity (beds available)

1.3 Experimental aims

A set of 72 indicative experimental parameter configurations were investigated:

Bed numbers between 30 and 70 beds (in increments of 5 beds; a total of 9 bed parameters) were investigated with each of two theatre schedules. The Baseline theatre schedule (4 theatres, 3 sessions per day: (i) revision *or* two primaries; (ii) revision *or* two primaries; (iii) one primary) with 5-day working. The Baseline + weekend schedule uses the same daily theatre allocations, and theatre numbers, with 7-day working. For each of these parameter changes, four scenarios were run for lengths-of-stay (baseline, 25% baseline) and for proportion delayed (baseline, 25% baseline). This totals 72 combinations. Reducing lengths-of-stay and proportion with a discharge delay by 75% brings them in line with national benchmarks. Other scenarios for increasing theatre capacity and changing theatre activity can additionally be investigated in the model.

2.0 Model logic
2.1 Base model overview diagram

For peer review only

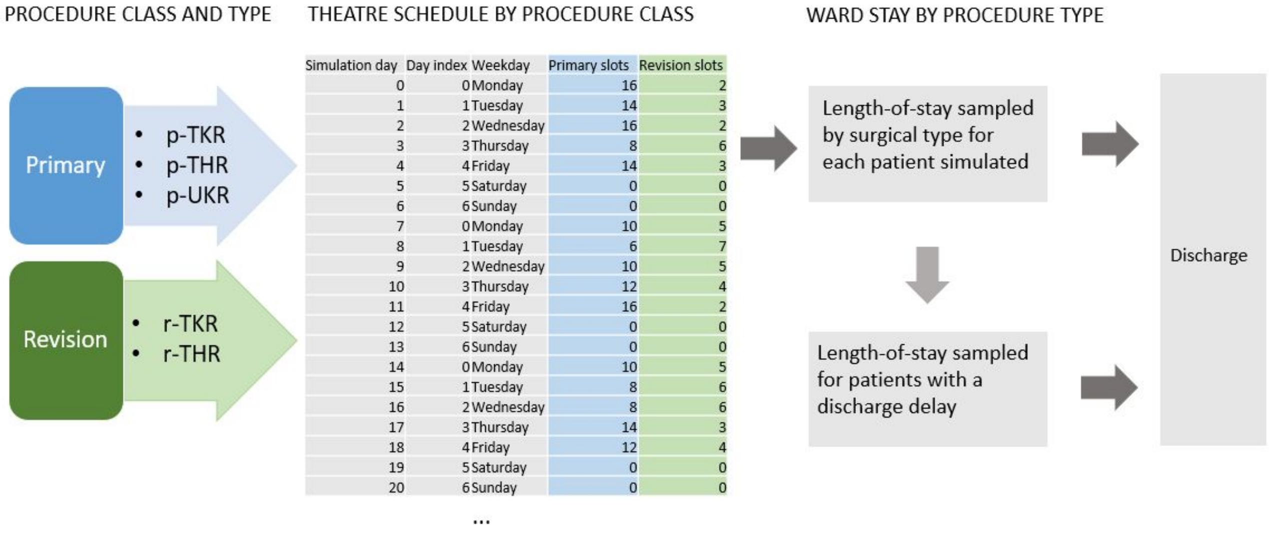

Simulation day	Day index	Weekday	Primary slots	Revision slots
0	0	Monday	16	2
1	1	Tuesday	14	3
2	2	Wednesday	16	2
3	3	Thursday	8	6
4	4	Friday	14	3
5	5	Saturday	0	0
6	6	Sunday	0	0
7	0	Monday	10	5
8	1	Tuesday	6	7
9	2	Wednesday	10	5
10	3	Thursday	12	4
11	4	Friday	16	2
12	5	Saturday	0	0
13	6	Sunday	0	0
14	0	Monday	10	5
15	1	Tuesday	8	6
16	2	Wednesday	8	6
17	3	Thursday	14	3
18	4	Friday	12	4
19	5	Saturday	0	0
20	6	Sunday	0	0

...

2.2 Base model logic

Patients arrive into the model deterministically using a pre-defined theatre schedule which allocates surgical classes (primary, revision) across a number of sessions per weekday, and a number of theatres per weekday. A surgical type is allocated by probability. Each surgical type samples a length-of-stay from a lognormal distribution fitted to historical data. A proportion of patients will have a delay to their discharge, for example waiting for out-of-hospital care. These patients have a length-of-stay sample from a lognormal distribution. If there is no bed available for a patient scheduled for surgery, this patient represents a 'lost slot'.

2.3 Scenario logic

- Patients arrive into the model according to a pre-defined theatre schedule. The schedule is an experimental parameter. Per weekday, the number of surgery sessions, the allocation of surgical classes per session, and the number of theatres available can be defined.
- Within the model which is made available in streamlit.io, the simulation parameters which can be changed are: number of beds; mean lengths-of-stay per surgical procedure; mean length-of-stay of patients whose discharge is delayed; proportion of patients whose discharge is delayed. Each simulation scenario can be run with the baseline theatre schedule alone, or additionally with a user-defined schedule.
- Within the model notebook, additional parameters can be changed. These include: stdev of all lengths of stay; proportions of surgical types; length-of-stay distributions.

2.4 Components

- A theatre schedule defines deterministic arrivals by patient class
- Patients renege after 0.5-1 day (uniform) if no bed is available ('lost slots')
- Each class has attributes associated with surgical procedure, or delayed discharge
- A single service node
- A single resource: beds
- FIFO queue discipline

3.0 Data

Routinely collected data from North Bristol NHS Trust was used to identify patients receiving elective joint arthroplasty between January 2016 and December 2019.

The Trust's electronic health records (EHR) were used to identify elective joint arthroplasty using a combination of OPCS4 procedure and surgical site codes. Five core elective orthopedic surgical procedure types were identified and verified. A small number of short day-case 'hip resurfacing' surgeries [n=52] were removed from the dataset. The five remaining surgical types were classified into two classes: (I) Primary (primary hip replacement [p-THR n=3057; 51%], primary knee replacement [p-TKR; n=2302; 38%], uni-compartmental knee replacement [p-UKR; n=679; 11%]); (II) Revision (revision hip replacement [r-THR; n=482; 55%], revision knee replacement [r-TKR; n=392; 45%]). Most patients did not remain in hospital once they were medically fit for discharge, however a proportion of patients in the EHR had a recorded medically fit for discharge date which preceded their actual discharge date (n=529; 7.6%).

A statistical probability distribution was fitted to each type of surgical procedure using the EHR data. The fitted length-of-stay parameters [procedure, mean days (u), standard deviation days (sd)] are: p-THR, u=4.4, sd=2.9 days; p-TKR u=4.7 days, sd=2.8; p-UKR u=2.9 days, sd=2.1; r-THR, u=6.9, sd=7.0; r-TKR, u=7.2, sd=7.6; delayed discharge, u=16.5; sd=15.1).

4.0 Experimentation
4.1 Initialisation

The model uses a warm-up period of 35 days based on visual inspection of outputs. The model is non-terminating.

4.2 Run Length

The model runs for 70 days. This can be changed by the user. Multiple independent replications are undertaken to control confidence intervals. The number of replications is determined using MORE plots, and is set to 50 by default.

Nelson, B. L. (2008) The MORE plot: Displaying measures of risk & error from simulation output. In *2008 Winter Simulation Conference* (pp. 413-416). IEEE.

5.0 Implementation
5.1 Software of programming language

The simulation was developed using Python 3.8. A conda software environment is used to manage versions on a local machine. Software versions are:

- jupyterlab=3.4.6
- jupyterlab-spellchecker=0.7.2
- matplotlib=3.5.3
- numpy=1.23.2
- pandas=1.4.4
- pip=22.2.2
- python=3.8.12
- scipy=1.9.1
- simpy=4.0.1
- streamlit==1.17.0

5.2 Random sampling
All sampling uses `numpy.random.Generator`. Common random number streams are used in the model, created through seed vectors (one seed for each activity in each replication).

5.3 Model execution
Simpy implements a process-based simulation worldview.

For peer review only

5.4 System specification
The model was run on Intel i7-12700H CPU with 32GB RAM running Pop!_OS 22.04 Linux.

6.0 Code access

Code, input parameters and output data used for results in this paper are available on GitHub: AliHarp/HEP: Model of orthopaedic ward (github.com)

They are permanently archived using Zenodo (HEP | Zenodo).

All are licensed using MIT permissive license MIT License | Choose a License.

For peer review only

Supplementary Material 2
Table S1: OPCS-4 codes used to identify hip and knee arthroplasty surgery

**PRIMARY TOTAL HIP REPLACEMENT**		
4 Character Code	Description	
W37.1	Primary total prosthetic replacement of hip joint using cement	
W37.8	Other specified total prosthetic replacement of hip joint using cement	
W37.9	Unspecified total prosthetic replacement of hip joint using cement	
W38.1	Primary total prosthetic replacement of hip joint not using cement	
W38.8	Other specified total prosthetic replacement of hip joint not using cement	
W38.9	Unspecified total prosthetic replacement of hip joint not using cement	
W39.1	Primary total prosthetic replacement of hip joint NEC	
W39.8	Other specified other total prosthetic replacement of hip joint	
W39.9	Unspecified other total prosthetic replacement of hip joint	
W43.1	Primary total prosthetic replacement of other joint using cement NEC	
W43.8	Other specified total prosthetic replacement of other joint using cement NEC	
W43.9	Unspecified total prosthetic replacement of other joint using cement NEC	
W44.1	Primary total prosthetic replacement of other joint not using cement NEC	
W44.8	Other specified total prosthetic replacement of other joint not using cement NEC	
W44.9	Unspecified total prosthetic replacement of other joint not using cement NEC	
W45.1	Other primary total prosthetic replacement of other joint NEC	
W45.8	Other specified total prosthetic replacement of other joint NEC	
W45.9	Unspecified total prosthetic replacement of other joint NEC	
W52.1	Primary prosthetic replacement of articulation of bone using cement NEC	
W52.8	Other specified prosthetic replacement of articulation of bone using cement NEC	
W52.9	Unspecified prosthetic replacement of articulation of bone using cement NEC	
W53.1	Primary prosthetic replacement of articulation of bone not using cement NEC	
W53.8	Other specified prosthetic replacement of articulation of bone not using cement NEC	

For peer review only

W53.9	Unspecified prosthetic replacement of articulation of bone not using cement NEC	
W54.1	Primary prosthetic replacement of articulation of bone NEC	
W54.8	Other specified prosthetic replacement of articulation of bone NEC	
W54.9	Unspecified prosthetic replacement of articulation of bone NEC	
W93.1	Primary hybrid prosthetic replacement of hip joint using cemented acetabular component	
W93.8	Other specified hybrid prosthetic replacement of hip joint using cemented acetabular component	
W93.9	Unspecified hybrid prosthetic replacement of hip joint using cemented acetabular component	
W94.1	Primary hybrid prosthetic replacement of hip joint using cemented femoral component	
W94.8	Other specified hybrid prosthetic replacement of hip joint using cemented femoral component	
W94.9	Unspecified hybrid prosthetic replacement of hip joint using cemented femoral component	
W95.1	Primary hybrid prosthetic replacement of hip joint using cement NEC	
W95.8	Other specified hybrid prosthetic replacement of hip joint using cement	
W95.9	Unspecified hybrid prosthetic replacement of hip joint using cement	
**RESURFACING / RECONSTRUCTION**		
4 Character Code	Description	
W58.1	Primary resurfacing arthroplasty of joint	
W58.8	Other specified reconstruction of joint	
W58.9	Unspecified other reconstruction of joint	
**LOCATION OF SURGERY**		
4 Character Code	Description	
Z75.6	Acetabulum	
Z76.1	Head of femur	
Z84.3	Hip joint	
**REVISION HIP REPLACEMENT**		
4 Character Code	Description	

For peer review only

W37.0	Conversion from previous cemented total prosthetic replacement of hip joint	
W37.2	Conversion to total prosthetic replacement of hip joint using cement	
W37.3	Revision of total prosthetic replacement of hip joint using cement	
W37.4	Revision of one component of total prosthetic replacement of hip joint using cement	
W38.0	Conversion from previous uncemented total prosthetic replacement of hip joint	
W38.2	Conversion to total prosthetic replacement of hip joint not using cement	
W38.3	Revision of total prosthetic replacement of hip joint not using cement	
W38.4	Revision of one component of total prosthetic replacement of hip joint not using cement	
W39.0	Conversion from previous total prosthetic replacement of hip joint NEC	
W39.2	Conversion to total prosthetic replacement of hip joint NEC	
W39.3	Revision of total prosthetic replacement of hip joint NEC	
W39.5	Revision of one component of total prosthetic replacement of hip joint NEC	
W43.0	Conversion from previous cemented total prosthetic replacement of joint NEC	+ Site code
W43.2	Conversion to total prosthetic replacement of joint using cement NEC	+ Site code
W43.3	Revision of total prosthetic replacement of joint using cement NEC	+ Site code
W43.4	Revision of one component of total prosthetic replacement of joint using cement NEC	+ Site code
W44.0	Conversion from previous uncemented total prosthetic replacement of joint NEC	+ Site code
W44.2	Conversion to total prosthetic replacement of joint not using cement NEC	+ Site code
W44.3	Revision of total prosthetic replacement of joint not using cement NEC	+ Site code
W44.4	Revision of one component of total prosthetic replacement of joint not using cement NEC	+ Site code
W45.0	Conversion from previous total prosthetic replacement of joint NEC	+ Site code
W45.2	Conversion to total prosthetic replacement of joint NEC	+ Site code
W45.3	Revision of total prosthetic replacement of joint NEC	+ Site code
W45.5	Revision of one component of total prosthetic replacement of joint NEC	+ Site code
W46.2	Conversion to prosthetic replacement of head of femur using cement	

For peer review only

W46.3	Revision of prosthetic replacement of head of femur using cement	
W47.2	Conversion to prosthetic replacement of head of femur not using cement	
W47.3	Revision of prosthetic replacement of head of femur not using cement	
W48.2	Conversion to prosthetic replacement of head of femur NEC	
W48.3	Revision of prosthetic replacement of head of femur NEC	
W52.0	Conversion from previous cemented prosthetic replacement of articulation of bone NEC	+ Site code
W52.2	Conversion to prosthetic replacement of articulation of bone using cement NEC	+ Site code
W52.3	Revision of prosthetic replacement of articulation of bone using cement NEC	+ Site code
W53.0	Conversion from previous uncemented prosthetic replacement of articulation of bone NEC	+ Site code
W53.2	Conversion to prosthetic replacement of articulation of bone not using cement NEC	+ Site code
W53.3	Revision of prosthetic replacement of articulation of bone not using cement NEC	+ Site code
W54.0	Conversion from previous prosthetic replacement of articulation of bone NEC	+ Site code
W54.2	Conversion to prosthetic replacement of articulation of bone NEC	+ Site code
W54.3	Revision of prosthetic replacement of articulation of bone NEC	+ Site code
W57.4	Conversion to excision arthroplasty of joint	+ Site code
W58.0	Conversion from previous resurfacing arthroplasty of joint	+ Site code
W58.2	Revision of resurfacing arthroplasty of joint	+ Site code
W93.0	Conversion from previous hybrid prosthetic replacement of hip joint using cemented acetabular component	
W93.2	Conversion to hybrid prosthetic replacement of hip joint using cemented acetabular component	
W93.3	Revision of hybrid prosthetic replacement of hip joint using cemented acetabular component	
W94.0	Conversion from previous hybrid prosthetic replacement of hip joint using cemented femoral component	
W94.2	Conversion to hybrid prosthetic replacement of hip joint using cemented femoral component	
W94.3	Revision of hybrid prosthetic replacement of hip joint using cemented femoral component	
W95.0	Conversion from previous hybrid prosthetic replacement of hip joint using cement NEC	
W95.2	Conversion to hybrid prosthetic replacement of hip joint using cement NEC	
W95.3	Revision of hybrid prosthetic replacement of hip joint using cement NEC	

For peer review only

**PRIMARY TOTAL KNEE REPLACEMENT**		
4 Character Code	Description	
W40.1	Primary total prosthetic replacement of knee joint using cement	
W40.8	Other specified total prosthetic replacement of knee joint using cement	
W40.9	Unspecified total prosthetic replacement of knee joint using cement	
W41.1	Primary total prosthetic replacement of knee joint not using cement	
W41.8	Other specified total prosthetic replacement of knee joint not using cement	
W41.9	Unspecified total prosthetic replacement of knee joint not using cement	
W42.1	Primary total prosthetic replacement of knee joint NEC	
W42.8	Other specified other total prosthetic replacement of knee joint	
W42.9	Unspecified other total prosthetic replacement of knee joint	
O18.1	Primary hybrid prosthetic replacement of knee joint using cement	
O18.8	Other specified hybrid prosthetic replacement of knee joint using cement	
O18.9	Unspecified hybrid prosthetic replacement of knee joint using cement	
**PRIMARY UNICONDYLAR / UNICOMPARTMENTAL KNEE OPERATIONS**		
4 Character Code	Description	
W52.1	Primary prosthetic replacement of articulation of bone using cement NEC	Require combination with site + combination codes to ID
W52.8	Other specified prosthetic replacement of articulation of other bone using cement	Require combination with site + combination codes to ID

For peer review only

W52.9	Unspecified prosthetic replacement of articulation of other bone using cement	Require combination with site + combination codes to ID
W53.1	Primary prosthetic replacement of articulation of bone not using cement NEC	Require combination with site + combination codes to ID
W53.9	Unspecified prosthetic replacement of articulation of other bone not using cement	Require combination with site + combination codes to ID
W54.0	Conversion from previous prosthetic replacement of articulation of bone NEC	Require combination with site + combination codes to ID
W54.1	Primary prosthetic replacement of articulation of bone NEC	Require combination with site + combination codes to ID
W54.8	Other specified other prosthetic replacement of articulation of other bone	Require combination with site + combination codes to ID
W54.9	Unspecified other prosthetic replacement of articulation of other bone	Require combination with site + combination codes to ID
W58.1	Primary resurfacing arthroplasty of joint	Require combination with site + combination codes to ID
**SITE OF SURGERY**		
Z76.5	Lower end of femur NEC	
Z77.4	Upper end of tibia NEC	
Z78.7	Patella	Will need care in extracting as PFJ replacement will be coded with this
Z84.4	Patellofemoral joint	Will need care in extracting as PFJ replacement will be coded with this
Z84.5	Tibiofemoral joint	
Z84.6	Knee joint	
**REVISION KNEE REPLACEMENT**		
4 Character Code	Description	
W40.0	Conversion from previous cemented total prosthetic replacement of knee joint	

For peer review only

W40.2	Conversion to total prosthetic replacement of knee joint using cement	
W40.3	Revision of total prosthetic replacement of knee joint using cement	
W40.4	Revision of one component of total prosthetic replacement of knee joint using cement	
W41.0	Conversion from previous uncemented total prosthetic replacement of knee joint	
W41.2	Conversion to total prosthetic replacement of knee joint not using cement	
W41.3	Revision of total prosthetic replacement of knee joint not using cement	
W41.4	Revision of one component of total prosthetic replacement of knee joint not using cement	
W42.0	Conversion from previous total prosthetic replacement of knee joint NEC	
W42.2	Conversion to total prosthetic replacement of knee joint NEC	
W42.3	Revision of total prosthetic replacement of knee joint NEC	
W42.4	Attention to total prosthetic replacement of knee joint NEC	Plus Y03.2 (Renewal of prosthesis in organ NOC) or Y03.7 (Removal of prosthesis from organ NOC)
W42.5	Revision of one component of total prosthetic replacement of knee joint NEC	
W42.6	Arthrolysis of total prosthetic replacement of knee joint	
W58.0	Conversion from previous resurfacing arthroplasty of joint	
W58.2	Revision of resurfacing arthroplasty of joint	Require combination with site + combination codes to ID
O18.0	Conversion from previous hybrid prosthetic replacement of knee joint using cement	
O18.2	Conversion to hybrid prosthetic replacement of knee joint using cement	
O18.3	Revision of hybrid prosthetic replacement of knee joint using cement	
O18.4	Attention to hybrid prosthetic replacement of knee joint using cement	
W52.0	Conversion from previous cemented prosthetic replacement of articulation of bone NEC	Require combination with site + combination codes to ID
W52.2	Conversion to prosthetic replacement of articulation of bone using cement NEC	Require combination with site + combination codes to ID

For peer review only

W52.3	Revision of prosthetic replacement of articulation of bone using cement NEC	Require combination with site + combination codes to ID
W53.0	Conversion from previous uncemented prosthetic replacement of articulation of bone NEC	Require combination with site + combination codes to ID
W53.2	Conversion to prosthetic replacement of articulation of bone not using cement NEC	Require combination with site + combination codes to ID
W53.3	Revision of prosthetic replacement of articulation of bone not using cement NEC	Require combination with site + combination codes to ID
W54.0	Conversion from previous prosthetic replacement of articulation of bone NEC	Require combination with site + combination codes to ID
W54.2	Conversion to prosthetic replacement of articulation of bone NEC	Require combination with site + combination codes to ID
W54.3	Revision of prosthetic replacement of articulation of bone NEC	Require combination with site + combination codes to ID
W54.4	Attention to prosthetic replacement of articulation of bone NEC	Require combination with site + combination codes to ID
W55.3	Conversion to prosthetic interposition arthroplasty of joint	Require combination with site + combination codes to ID
W56.4	Conversion to interposition arthroplasty of joint NEC	Require combination with site + combination codes to ID
W57.4	Conversion to excision arthroplasty of joint	Require combination with site + combination codes to ID
W60.3	Conversion to arthrodesis and extra-articular bone graft NEC	Require combination with site + combination codes to ID
W61.3	Conversion to arthrodesis and articular bone graft NEC	Require combination with site + combination codes to ID
W64.1	Conversion to arthrodesis and internal fixation NEC	Require combination with site + combination codes to ID
W64.2	Conversion to arthrodesis and external fixation NEC	Require combination with site + combination codes to ID

For peer review only

BMJ Open

Development and application of simulation modelling for orthopaedic elective resource planning in England

Journal:	*BMJ Open*
Manuscript ID	bmjopen-2023-076221.R1
Article Type:	Original research
Date Submitted by the Author:	08-Nov-2023
Complete List of Authors:	Harper, Alison; NIHR Applied Research Collaboration South West Peninsula, University of Exeter Medical School; University of Exeter Faculty of Health and Life Sciences<br>Monks, Thomas; NIHR Applied Research Collaboration South West Peninsula, University of Exeter Medical School; University of Exeter Faculty of Health and Life Sciences<br>Wilson, Rebecca; NIHR Applied Research Collaboration West; University of Bristol, Population Health Sciences<br>Redaniel, Maria Theresa; NIHR Applied Research Collaboration West; University of Bristol, Population Health Sciences<br>Eyles, Emily; NIHR Applied Research Collaboration West; University of Bristol, Population Health Sciences<br>Jones, Tim; NIHR Applied Research Collaboration West; University of Bristol, Population Health Sciences<br>Penfold, Chris; NIHR Bristol Biomedical Research Centre; University of Bristol<br>Elliott, Andrew; North Bristol NHS Trust Southmead Hospital<br>Keen, Tim; North Bristol NHS Trust Southmead Hospital<br>Pitt, Martin; NIHR Applied Research Collaboration South West Peninsula; University of Exeter Faculty of Health and Life Sciences<br>Blom, Ashley; The University of Sheffield<br>Whitehouse, Michael; North Bristol NHS Trust Southmead Hospital<br>Judge, Andrew; NIHR Bristol Biomedical Research Centre; University of Bristol
**Primary Subject Heading**:	Health services research
Secondary Subject Heading:	Research methods
Keywords:	ORTHOPAEDIC & TRAUMA SURGERY, Information management < BIOTECHNOLOGY & BIOINFORMATICS, World Wide Web technology < BIOTECHNOLOGY & BIOINFORMATICS, Organisation of health services < HEALTH SERVICES ADMINISTRATION & MANAGEMENT, Orthopaedic & trauma surgery < SURGERY

SCHOLARONE™
Manuscripts

I, the Submitting Author has the right to grant and does grant on behalf of all authors of the Work (as defined in the below author licence), an exclusive licence and/or a non-exclusive licence for contributions from authors who are: i) UK Crown employees; ii) where BMJ has agreed a CC-BY licence shall apply, and/or iii) in accordance with the terms applicable for US Federal Government officers or employees acting as part of their official duties; on a worldwide, perpetual, irrevocable, royalty-free basis to BMJ Publishing Group Ltd ("BMJ") its licensees and where the relevant Journal is co-owned by BMJ to the co-owners of the Journal, to publish the Work in this journal and any other BMJ products and to exploit all rights, as set out in our licence.

The Submitting Author accepts and understands that any supply made under these terms is made by BMJ to the Submitting Author unless you are acting as an employee on behalf of your employer or a postgraduate student of an affiliated institution which is paying any applicable article publishing charge ("APC") for Open Access articles. Where the Submitting Author wishes to make the Work available on an Open Access basis (and intends to pay the relevant APC), the terms of reuse of such Open Access shall be governed by a Creative Commons licence – details of these licences and which Creative Commons licence will apply to this Work are set out in our licence referred to above.

Other than as permitted in any relevant BMJ Author's Self Archiving Policies, I confirm this Work has not been accepted for publication elsewhere, is not being considered for publication elsewhere and does not duplicate material already published. I confirm all authors consent to publication of this Work and authorise the granting of this licence.

For peer review only

DEVELOPMENT AND APPLICATION OF SIMULATION MODELLING FOR ORTHOPAEDIC ELECTIVE RESOURCE PLANNING IN ENGLAND

Alison Harper[1], Thomas Monks[1], Rebecca Wilson[2], Maria Theresa Redaniel[2], Emily Eyles[2], Tim Jones[2], Chris Penfold[3], Andrew Elliott[4], Tim Keen[4], Martin Pitt[1], Ashley Blom[5], Michael Whitehouse[4], and Andrew Judge[3]

[1]The National Institute for Health and Care Research Applied Research Collaboration West (NIHR PenARC) at University of Exeter a.l.harper@exeter.ac.uk t.m.w.monks@exeter.ac.uk m.pitt@exeter.ac.uk

[2] The National Institute for Health and Care Research Applied Research Collaboration West (NIHR ARC West) at University Hospitals Bristol and Weston NHS Foundation Trust, UK; Population Health Sciences, Bristol Medical School, University of Bristol, UK Rebecca.wilson@bristol.ac.uk Theresa.redaniel@bristol.ac.uk Emily.eyles@bristol.ac.uk timothy.jones@bristol.ac.uk

[3]NIHR Biomedical Research Centres University Hospitals Bristol and Weston NHS FT and University of Bristol Andrew.judge@bristol.ac.uk chris.penfold@bristol.ac.uk

[4]North Bristol NHS Foundation Trust tim.keen@nbt.nhs.uk Andrew.elliott@nbt.nhs.uk Michael.Whitehouse@bristol.ac.uk

[5]Faculty of Health, University of Sheffield a.blom@sheffield.ac.uk

Correspondence to Alison Harper: a.l.harper@exeter.ac.uk

Keywords: Orthopedics, Benchmarking, Mobile Applications, Simulation, Elective

Word count: 3658

ABSTRACT

Objectives: To develop a simulation model to support orthopaedic elective capacity planning.

Methods: An open-source, generalisable discrete-event simulation was developed, including a web-based application. The model used anonymised patient records between 2016-2019 of elective orthopaedic procedures from an NHS Trust in England. In this paper, it is used to investigate scenarios including resourcing (beds and theatres) and productivity (lengths-of-stay, delayed discharges, theatre activity) to support planning for meeting new NHS targets aimed at reducing elective orthopaedic surgical backlogs in a proposed ring fenced orthopaedic surgical facility. The simulation is interactive and intended for use by health service planners and clinicians.

Results: A higher number of beds (65-70) than the proposed number (40 beds) will be required if lengths-of-stay and delayed discharge rates remain unchanged. Reducing lengths-of-stay in line with national benchmarks reduces bed utilisation to an estimated 60%, allowing for additional theatre activity such as weekend working. Further, reducing the proportion of patients with a delayed discharge by 75% reduces bed utilisation to below 40%, even with weekend working. A range of other scenarios can also be investigated directly by NHS planners using the interactive web app.

Conclusions: The simulation model is intended to support capacity planning of orthopaedic elective services by identifying a balance of capacity across theatres and beds and predicting the impact of productivity measures on capacity requirements. It is applicable beyond the study site and can be adapted for other specialties.

Strengths and Limitations of this study:

For peer review only

- The simulation model provides rapid quantitative estimates to support post-COVID elective services recovery toward medium-term elective targets.
- Parameter combinations include changes to both resourcing and productivity.
- The interactive web app enables meaningful and relevant parameter changes by healthcare managers, planners, and clinicians.
- Patient attributes such as complexity are not included in the model but are reflected in variables such as length-of-stay and delayed discharge rates.
- Theatre schedules are simplified, incorporating the five key orthopaedic elective surgical procedures.

INTRODUCTION

Elective joint replacement comprises one of the highest volumes of elective procedures worldwide [1]. In the UK, orthopaedics has been the specialty under most pressure in terms of performance against National Health Service (NHS) elective operational standards [2]. Prior to the COVID-19 pandemic, increased waiting times for elective orthopaedic surgery reflected limited NHS resources and the competing demands of rising emergency admissions that affect mixed sites. This has been particularly problematic during the winter months when emergency demand for hospital care is high [3].

Hip and knee replacement have been shown to be strongly cost effective from both a societal and a health system perspective compared to non-surgical treatment [4]. Procedures that are not delayed are more cost effective than delayed intervention, while patients delayed for surgery for more than 180 days have been shown to be at higher risk of poor outcomes [4-6]. Additionally, reviews have found that low surgical volume is associated with longer procedure times and lengths-of-stay, and poorer patient outcomes including risk of revision [7-9]. Given substantial evidence that surgeons undertaking low volumes of specific surgical activities may increase costs and result in less favourable outcomes for patients, Getting it Right First Time (GIRFT) [10] published a set of recommendations, such as ring-fencing beds and improving criteria-led discharge, aimed at reducing the significant variation found in practice. While successful [11], performance against core national standards have continued to deteriorate, attributable to increasing demand and lack of available capacity [2, 12].

The effect of the COVID-19 pandemic on elective orthopaedic services has been to compound ongoing challenges, with larger waiting lists and a steep decline in performance. Following the pandemic, a deterioration has been found in the health of patients who have had elective joint replacement postponed [13]. In response to this situation, the government has committed two-year revenue allocations to support Integrated Care Boards (ICBs) to expand capacity. ICBs and primary and secondary care providers are required to develop plans to meet national objectives and local priorities, in particular to eliminate elective waits of over 65 weeks by March 2024 [14]. While central capital funding will be key to achieving this, maximising use of resources and reducing lengths-of-stay and delayed discharges are required to make effective use of available capacity, and are associated with improvements in patient care [12].

ICBs are therefore currently working to secure capital funds by delivering business cases that evidence optimal capacity and productivity configurations considering activity, workforce, capital requirements and potential revenue. Simulation modelling can be used as a planning tool to provide supporting evidence by modelling various configurations of bed numbers, theatre capacity, and ward stays to estimate resultant surgical throughput. In this paper, we describe the development and implementation of an interactive, free and open-source simulation model to support planning of ring-fenced elective orthopaedic capacity. The model is co-designed with North Bristol NHS Trust (NBT), and is designed to be reusable, generalisable and to provide rapid information for clinicians, business and service managers across a range of scenarios relevant to new orthopaedic capacity planning.

For peer review only

METHODS

We developed a discrete-event simulation (DES) model (programmed in Python 3.8) of surgical activity and ward stay in a proposed ring-fenced orthopaedic facility. DES allows processes and pathways to be modelled at individual patient level, and to explore the potential impact of changes to the system without the costs and risks associated with real-world changes. It has been used for patient flow management, resource allocation, and scheduling, for example in sexual health [15], stroke pathways [16], and orthopaedics [17-18]. The DES was developed to have the flexibility to answer a range of 'what-if' questions of interest to NBT, and is generalisable to other NHS Trusts for orthopaedic elective planning.

The DES model is free and open source, and is available as a web app: https://hospital-efficiency-project.streamlit.app/. To preserve code, we have permanently archived it using Zenodo (HEP | Zenodo) [19]. All code has an MIT license allowing free reuse and adaptation by researchers, industry and the NHS. Our app provides a user-friendly, interactive interface for the DES, including instructions for use and documentation, allowing NHS staff to experiment with model parameters and generate immediate results without the need to download and install software. The model can also be adapted to other specialties.

The model is documented using STRESS reporting guidelines [20], available in supplementary material 1.

Data and Setting

NBT serves a population of approximately 1-million people, with an age-profile in line with England. Routinely collected data from the NHS Trust was used to identify patients receiving elective joint replacement between January 2016 and December 2019.

The Trust's electronic health records (EHR) were used to identify elective joint replacements using a combination of OPCS4 procedure and surgical site codes (Supplementary material 2). Five core elective orthopedic surgical procedure types were identified and verified. A small number of short day-case 'hip resurfacing' surgeries [n=52] were removed from the dataset as they rarely utilize bed capacity. The five remaining surgical types were classified into two classes: (I) *Primary [87%]:* (primary hip replacement [p-THR n=3057; 51%], primary knee replacement [p-TKR; n=2302; 38%], uni-compartmental knee replacement [p-UKR; n=679; 11%]); (II) *Revision [13%]:* (revision hip replacement [r-THR; n=482; 55%], revision knee replacement [r-TKR; n=392; 45%]). Most patients did not remain in hospital once they were medically fit for discharge, however a proportion of patients in the EHR had a recorded *medically fit for discharge* date which preceded their actual discharge date (n=529; 7.6%).

The DES requires parameters describing patient lengths-of-stay, hence statistical probability distributions were fitted to each category of surgical procedure using the EHR data. The length-of-stay parameters [procedure, mean days (u), standard deviation days (sd)] are: p-THR, u=4.4, sd=2.9; p-TKR, u=4.7, sd=2.8; p-UKR, u=2.9, sd=2.1; r-THR, u=6.9, sd=7.0; r-TKR, u=7.2, sd=7.6; delayed discharge, u=16.5; sd=15.1), which are converted to lognormal parameters within the model. Lognormal distributions were used for sampling lengths-of-stay in all cases. The mean lengths-of-stay are high against national benchmarks, and a key focus of future activity is to reduce lengths-of-stay [11, 21].

Orthopaedic surgical pathway

The DES model is a simplified, high-level representation of the system of interest, which simulates individual patient flow through the system over time. Our model assumes an infinite waiting list. Baseline surgical theatre scheduling rules define how patients enter the simulation model according to their surgical class (Primary or Revision). Baseline rules are:

- Three theatre sessions per day, five days per week with no weekend activity.
- Morning and afternoon sessions schedule either: one Revision *or* two Primary surgeries.
- Evening session schedules one Primary surgery.

For peer review only

Baseline resources for the model are 40 beds and 4 theatres. The DES samples a length-of-stay for each simulated patient from a lognormal distribution with parameters calculated from the previously fitted distributions by procedure type. Using stochastic routing, a length-of-stay is sampled for delayed patients. The simulation can be run using baseline parameters, and additional scenarios can be run by changing these parameters to determine the effects on model outputs, primarily surgical throughput. Figure 1 shows the organisation of surgical activity and ward stay.

Figure 1: Conceptual organisation of ring-fenced orthopaedic activity and ward stay

Model objectives

With the overall objective of maximising surgical throughput, the effects of changing model inputs can be investigated alone or in combination as experimental scenarios. These include:

- The number of theatres;
- The number of ring-fenced beds;
- Patient lengths-of-stay, including delayed discharges;
- Proportion of patients with a delayed discharge;
- Effects of running evening or weekend theatre sessions;
- Changes to surgical scheduling, for example scheduling revision surgery (with longer, more variable lengths-of-stay) earlier in the week.

These questions are all in line with GIRFT priorities, which include accepting day surgery as default (where a bed is available as back-up), improving theatre utilisation and best practice care, and focused enhanced recovery. Higher surgical volumes, dedicated theatre teams, and enhanced post-operative recovery is expected to improve patient outcomes. In turn, it is expected that lengths-of-stay will reduce, and standardised clinical pathways and discharge planning are likely to reduce discharge delays [12, 21].

The model outputs:

- *Total surgical throughput*. The primary goal of central capital funding allocations is to efficiently maximize surgical throughput [14], so the configuration which best achieves this - within other constraints relevant to service planners such as workforce availability [22] - is a key output.
- *Bed utilisation per day of week.* For each experimental scenario, mean bed utilisation (occupancy) is outputted daily over model runtime. While there is no ideal average bed utilisation figure (which is dependent upon many factors), it is commonly accepted that mean occupancies greater than 85-90% can expect regular bed crises [23]. The results of excessive bed utilisation in the model can be seen as 'lost slots', where no bed is available for a patient scheduled for surgery. An additional consideration is that GIRFT recommend the extension of therapy services to support patient mobility goals toward discharge on any day with elective operating [21]. Therefore, theatre scheduling decisions (e.g., 5-7 day service) are dependent upon the availability of weekend staff.
- *Lost theatre slots for system reasons*. While beds are protected from outlying emergency admissions in a ring-fenced scenario, the balance of beds to theatre activity is a critical question. In the model, where patients are scheduled to arrive for surgery but no bed is available, the theatre slot is lost. In reality, other system behaviours will account for some of these lost slots. For example, the slot may be lost for patient reasons such as illness (i.e. the patient doesn't attend for surgery or is deemed not fit for surgery at the point of admission); bed management activities may free up beds; or patients may be transferred to the acute hospital. In the model, lost slots (per day of week) are an indication of a mismatch between demand (theatre scheduling) and capacity (bed utilisation). In NBT, an average of 4.75 slots are lost per week for patient reasons, and a further 2.5 for system reasons.

For peer review only

Patient and public involvement

Patients and the public were consulted in a workshop for suggestions and comments to inform the development of the grant that supported this work, and a further workshop informed scenarios used in model development.

Role of the funding source

The funders had no role in any of the following: the study design; the collection, analysis, and interpretation of data; the writing of the report; the decision to submit the paper for publication.

RESULTS

A set of 72 indicative experimental parameter configurations were investigated (summarised in Table 1) and described here:

Bed numbers between 30 and 70 beds (in increments of 5 beds; a total of 9 bed parameters) were investigated with each of two theatre schedules. The Baseline theatre schedule is as described in the Methods section, with 5-day working. The Baseline + weekend schedule uses the same daily theatre allocations and theatre numbers, with 7-day working. For each of these parameter changes, four scenarios were run for lengths-of-stay (baseline, 25% baseline) and for proportion delayed (baseline, 25% baseline). This totals 72 combinations.

Table 1: Summary of scenarios varying procedure lengths-of-stay (los), bed numbers, proportion of patients with a delayed discharge (prop), and daily theatre schedule.

Scenario	Length-of-stay (los): 2 parameters	Proportion delayed (prop): 2 parameters	Beds: 9 parameters	Schedule: 2 parameters
Scenario 1	Baseline	Baseline	30 – 70 (in intervals of 5)	Baseline<br>Baseline + weekend
Scenario 2	0.25x Baseline	0.25x Baseline	30 – 70 (in intervals of 5)	Baseline<br>Baseline + weekend
Scenario 3	Baseline	0.25x Baseline	30 – 70 (in intervals of 5)	Baseline<br>Baseline + weekend
Scenario 4	0.25x Baseline	Baseline	30 – 70 (in intervals of 5)	Baseline<br>Baseline + weekend

The baseline procedure lengths-of-stay and proportion of patients with a delayed discharge are historical values described in the Methods section. As a goal of capacity planning is to reduce lengths-of-stay and delayed discharge, these are considered maximum values (high_los, prop_high), with minimum values set at 25% of baseline (low_los, prop_low). In all cases, the length-of-stay parameters for those patients with a discharge delay remain at the baseline value.

The results are plotted in Figure 2. The top row displays the mean total daily surgical throughput for each scenario, and for each theatre schedule. The middle row is mean daily bed utilisation. The bottom row displays 'lost slots', estimating the extent of the mismatch between patients scheduled and beds available.

Figure 2: Results of simulations across 30-70 beds, for each of 2 theatre schedules with 4 combinations of lengths-of-stay (baseline:high_los; baseline x0.25: low_los) and proportion delayed (baseline:prop_high; baseline x0.25: prop_los) for mean daily total surgical throughput, bed utilisation, and lost slots.

The results show that the system is more sensitive to changes in procedure lengths-of-stay than to changes in the proportion of patients delayed, despite the long mean lengths-of-stay for delayed patients. At current procedure lengths-of-stay (high_los), bed utilisation is high with both current and reduced delayed discharges

For peer review only

(Scenarios 1 and 3). With reduced lengths-of-stay, the effects of reducing delayed discharges on required bed numbers is more significant, substantially reducing bed utilisation.

In the case of no weekend activity, a higher number of beds (65-70) than the proposed value (40 beds) will be required if lengths-of-stay remain unchanged. At this level of bed utilisation, reducing delayed discharges has little impact on required bed numbers. However, reducing lengths-of-stay in line with national benchmarks has enough impact on bed utilisation to allow for additional theatre activity.

Where lengths-of-stay can be reduced, weekend operating theatre activity (remaining at 4 theatres) increases surgical throughput, and beds remain underutilised in all cases above 40 beds. Reducing the proportion of patients with delayed discharge further reduces bed requirements to approximately 30 beds. Where lengths-of-stay remain at baseline values, weekend theatre activity cannot be considered, as bed utilisation and resultant lost slots are unacceptably high, even up to 70 beds. Users can investigate scenarios between these extreme values to gain realistic expectations of required bed and theatre numbers. Within the web application, results for bed utilisation include variability in output using boxplots. Sensitivity analysis and testing of parameters has been undertaken and is available to view in GitHub, Zenodo [19], and our online notebook (see Data Availability Statement).

DISCUSSION

Our generalisable, open access application allows those involved in planning the development and utilisation of ring-fenced elective orthopaedic units to rapidly model different scenarios to predict delivery of elective surgical care. The model is adaptable to use local data for other units to model likely scenarios when planning activity. With minimal adaptation, it can also be applied to the delivery of other types of elective surgery.

Our experimental scenarios found that reducing lengths-of-stay in line with national benchmarks can increase surgical throughput and allow for additional theatre activity given the bed and theatre numbers initially proposed. We investigated weekend working, but the model also allows estimates to be obtained for increased theatre numbers, increased theatre utilisation (more procedures per day), and changes to theatre activity (e.g., scheduling more complex surgery early in the week). However, if lengths-of-stay remain unchanged, proposed bed numbers in this instance will be inadequate. As procedure lengths-of-stay reduce, the effects of reducing the number of patients with a delayed discharge also become more significant. Reducing the length of the delay will similarly reduce bed utilisation and increase throughput, and the results of this can also be investigated using the simulation model.

A strength of simulation modelling is the use of underlying stochastic distributions using real-world data, as planning by average occupancy will not provide adequate reserve capacity to manage the variation seen in patient lengths-of-stay [24]. Our open approach to modeling is a further strength, as a range of scenario combinations can be investigated by users to support planning using the web app. Additionally, the model is available for re-use either through re-parameterisation or adaptation. There are limitations to the use of simulation modeling. Assumptions and simplifications are required to convert a real-world problem into a computer representation. We assume that all historical (pre-pandemic) lengths-of-stay distributions fit current lengths-of-stay for baseline modelling. We do not account for patient frailty or other patient attributes beyond length-of-stay and the proportion of delayed discharges, and it is possible that patients who have been delayed are more complex. Additionally, our theatre scheduling rules include only the five main elective orthopaedic procedures. While other procedures may utilize theatre activity, they are not accounted for in bed planning, although simpler procedures will more likely to be performed as day cases, and more complex procedures (such as spinal surgery) will be performed in a main hospital setting with high dependency facilities.

Previous modelling and simulation work has focused on resource sharing for elective and non-elective joint replacement [17] and detailed studies of individual orthopaedic services [18, 25]. Although the need for reusable models for orthopaedic wait list planning is recognised [26], this is the first free and open-source, generalisable DES model to support resourcing and efficiency of elective services that is available for use in

For peer review only

this area [27]. Our model provides quantitative outputs estimating the effects on surgical throughput (per procedure); daily bed utilisation of changes to bed and theatre capacity, theatre scheduling, patient lengths-of-stay and discharge delays; and lost theatre slots, representing system pressure on beds. The model is designed for use by health services planners and clinicians, and is available as a free web-app to address usability and accessibility of results [28]. It is being used to evidence service configurations for the business case in NBT, and is more widely applicable.

CONCLUSIONS

Post-pandemic, pressure to restore elective surgeries against new interim national targets necessitates efficient and effective use of allocated public funds. This planning is happening rapidly, and on a large scale across the UK. Simulation modelling offers an effective method for planning elective services, identifying a balance of capacity across theatres and beds, and predicting the impact of productivity measures on capacity requirements. The model developed in this study is being used to provide quantitative support for accessing central capital funds, enabling discussion and evidence for the most efficient use of new resourcing. The model has been developed to offer a transferable solution for supporting both orthopaedic elective recovery, and with minor adaptations, recovery of other elective services.

Data availability statement

Data may be obtained from a third party and are not publicly available. The data used in the study are collected by the North Bristol NHS Trust (NBT) as part of their care and support. Sharing of anonymised data with the University of Bristol was underpinned by a data sharing agreement and solely covers the purposes of this study. Data requests can be made through the NBT.

Code, input parameters and output data used for results in this paper are available on GitHub: https://github.com/AliHarp/HEP and archived using Zenodo https://zenodo.org/records/7951080. Code, results, and testing can be viewed at https://aliharp.github.io/HEP/HEP_notebooks/01_intro.html. All are licensed using MIT permissive license MIT License | Choose a License.

Patient consent for publication

Not applicable.

Ethics approval

We were provided with pseudonymised North Bristol Trust hospital admissions data under the NIHR ARC West Partnership Agreement. The project received ethical approval from the University of Bristol Faculty of Health Sciences ethical review board on 3 November 2020 (ref# 109024).

Funding

This study was funded by the Health Data Research (HDR) UK South West Better Care Partnership (#6.12). AH, TM and MP are supported by the National Institute for Health Research Applied Research Collaboration South West Peninsula. RW, EE, TJ and MTR's time was supported by the National Institute for Health Research Applied Research Collaboration West (NIHR ARC West). AJ, MW, AB and CP were supported by the NIHR Biomedical Research Centre at University Hospitals Bristol and Weston NHS Foundation Trust and the University of Bristol. The views expressed in this publication are those of the author(s) and not necessarily those of the National Institute for Health Research or the Department of Health and Social Care.

Competing interests None declared

Contributors AH drafted the original manuscript. AH, TM, and MP developed the model. AH, TM, RW, MTR, EE, TJ, CP, AE, TK, MP, AB, MW, and AJ contributed to the conception and planning of this project, the acquisition of data, critically reviewed and revised the manuscript, and approved the final version. MTR

For peer review only

and AJ supervised the project. MTR is the guarantor for the project. All authors agree to be accountable for all aspects of the work.

REFERENCES

[1] Meara, J.G., Leather, A.J., Hagander, L., Alkire, B.C., Alonso, N., Ameh, E.A., Bickler, S.W., Conteh, L., Dare, A.J., Davies, J. and Mérisier, E.D., 2015. Global Surgery 2030: evidence and solutions for achieving health, welfare, and economic development. *The lancet*, *386*(9993), pp.569-624.

[2] The Kings Fund. 2021. Waiting times for elective (non-urgent) treatment: referral to treatment (RTT) Available at: Waiting times for elective (non-urgent) treatment: referral to treatment (RTT) | The King's Fund (kingsfund.org.uk)

[3] Alderson, D., 2018. Winter pressures that last all year. *The Bulletin of the Royal College of Surgeons of England*, *100*(2), pp.53-53.

[4] Lan, R.H., Yu, J., Samuel, L.T., Pappas, M.A., Brooks, P.J. and Kamath, A.F., 2020. How are we measuring cost-effectiveness in total joint arthroplasty studies? Systematic review of the literature. *The Journal of arthroplasty*, *35*(11), pp.3364-3374.

[5] Kapstad, H., Rustøen, T., Hanestad, B.R., Moum, T., Langeland, N. and Stavem, K., 2007. Changes in pain, stiffness and physical function in patients with osteoarthritis waiting for hip or knee joint replacement surgery. *Osteoarthritis and cartilage*, *15*(7), pp.837-843.

[6] Scott, C.H., MacDonald, D.J. and Howie, C.R., 2019. 'Worse than death' and waiting for a joint arthroplasty. *The bone & joint journal*, *101*(8), pp.941-950.

[7] Lau, R.L., Perruccio, A.V., Gandhi, R. and Mahomed, N.N., 2012. The role of surgeon volume on patient outcome in total knee arthroplasty: a systematic review of the literature. *BMC musculoskeletal disorders*, *13*(1), pp.1-10.

[8] Pamilo, K.J., Peltola, M., Mäkelä, K., Häkkinen, U., Paloneva, J. and Remes, V., 2013. Is hospital volume associated with length-of-stay, re-admissions and reoperations for total hip replacement? A population-based register analysis of 78 hospitals and 54,505 replacements. *Archives of orthopaedic and trauma surgery*, *133*, pp.1747-1755.

[9] Sayers, A., Steele, F., Whitehouse, M.R., Price, A., Ben-Shlomo, Y. and Blom, A.W., 2020. Association between surgical volume and failure of primary total hip replacement in England and Wales: findings from a prospective national joint replacement register. *BMJ open*, *10*(9), p.e033045.

[10] GIRFT. 2015. A national review of adult elective orthopaedic services in England GETTING IT RIGHT FIRST TIME. Available at: Layout 1 (gettingitrightfirsttime.co.uk)

[11] GIRFT. 2020. Getting it Right in Orthopaedics: A follow-up on the GIRFT national specialty report on orthopaedics. Available at: Layout 1 (gettingitrightfirsttime.co.uk)

[12] Wall, J., Ray, S. and Briggs, T.W., 2022. Delivery of elective care in the future. *Future healthcare journal*, *9*(2), p.144.

[13] Pietrzak, J.R.T., Maharaj, Z., Erasmus, M., Sikhauli, N., Cakic, J.N. and Mokete, L., 2021. Pain and function deteriorate in patients awaiting total joint arthroplasty that has been postponed due to the COVID-19 pandemic. *World Journal of Orthopedics*, *12*(3), p.152.

[14] NHS. 2022. Delivery Plan for Tackling the Covid-19 backlog of elective care. Available at: https://www.england.nhs.uk/coronavirus/wp-content/uploads/sites/52/2022/02/C1466-delivery-plan-for-tackling-the-covid-19-backlog-of-elective-care.pdf

For peer review only

[15] Mohiuddin, S., Gardiner, R., Crofts, M., Muir, P., Steer, J., Turner, J., Wheeler, H., Hollingworth, W. and Horner, P.J., 2020. Modelling patient flows and resource use within a sexual health clinic through discrete event simulation to inform service redesign. *BMJ open*, *10*(7), p.e037084.

[16] Allen, M., Pearn, K., Monks, T., Bray, B.D., Everson, R., Salmon, A., James, M. and Stein, K., 2019. Can clinical audits be enhanced by pathway simulation and machine learning? An example from the acute stroke pathway. *BMJ open*, *9*(9), p.e028296.

[17] Persson, M., Hvitfeldt-Forsberg, H., Unbeck, M., Sköldenberg, O.G., Stark, A., Kelly-Pettersson, P. and Mazzocato, P., 2017. Operational strategies to manage non-elective orthopaedic surgical flows: a simulation modelling study. *BMJ open*, *7*(4), p.e013303.

[18] Anderson, G.H., Jenkins, P.J., McDonald, D.A., Van Der Meer, R., Morton, A., Nugent, M. and Rymaszewski, L.A., 2017. Cost comparison of orthopaedic fracture pathways using discrete event simulation in a Glasgow hospital. *BMJ open*, *7*(9), p.e014509.

[dataset] [19] Harper, A., 2023. Code and materials for BMJ Open submission of Hospital Efficiency Project orthopaedic planning DES. Zenodo, 19 May 2023. HEP: BMJ Open release. https://zenodo.org/records/7951080

[20] Monks, T., Currie, C.S., Onggo, B.S., Robinson, S., Kunc, M. and Taylor, S.J., 2019. Strengthening the reporting of empirical simulation studies: Introducing the STRESS guidelines. *Journal of Simulation*, *13*(1), pp.55-67.

[21] GIRFT. 2023. Orthopaedic Elective Surgery Guide to delivering perioperative ambulatory care for patients with hip and knee pain requiring joint replacement surgery. Available at: SWAOC-Hip-and-Knee-Delivery-Guide-March-2023-FINAL-V1-1.pdf (gettingitrightfirsttime.co.uk)

[22] Mahase, E., 2022. Health leaders question absence of workforce strategy in NHS elective care recovery plan. https://doi.org/10.1136/bmj.o343

[23] Bain, C.A., Taylor, P.G., McDonnell, G. and Georgiou, A., 2010. Myths of ideal hospital occupancy. *Medical Journal of Australia*, *192*(1), pp.42-43.

[24] Monks, T., Worthington, D., Allen, M., Pitt, M., Stein, K. and James, M.A., 2016. A modelling tool for capacity planning in acute and community stroke services. *BMC health services research*, *16*(1), pp.1-8.

[25] Baril, C., Gascon, V. and Cartier, S., 2014. Design and analysis of an outpatient orthopaedic clinic performance with discrete event simulation and design of experiments. *Computers & Industrial Engineering*, *78*, pp.285-298.

[26] Boyle, L. and Mackay, M., 2022, December. A Reusable Discrete Event Simulation Model for Improving Orthopedic Waiting Lists. In *2022 Winter Simulation Conference (WSC)* (pp. 973-984). IEEE.

[27] Monks, T. and Harper, A., 2023. Computer model and code sharing practices in healthcare discrete-event simulation: a systematic scoping review. *Journal of Simulation*, 10.1080/17477778.2023.2260772

[28] Monks T. and Harper, A., 2023. Improving the usability of open health service delivery simulation models using Python and web apps. *NIHR Open Res*. 3(48) 10.3310/nihropenres.13467.1

For peer review only

Figure 1: Conceptual organisation of ring-fenced orthopaedic activity and ward stay

Figure 2: Results of simulations across 30-70 beds, for each of 2 theatre schedules with 4 combinations of lengths-of-stay (baseline:high_los; baseline x0.25: low_los) and proportion delayed (baseline:prop_high; baseline x0.25: prop_los) for mean daily total surgical throughput, bed utilisation, and lost slots.

For peer review only

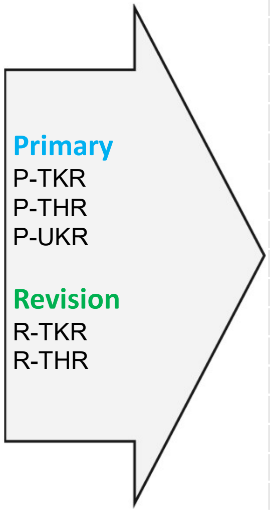

Simulation day	Day index	Weekday	Primary	Revision
0	0	Monday	16	2
1	1	Tuesday	14	3
2	2	Wednesday	16	2
3	3	Thursday	8	6
4	4	Friday	14	3
5	5	Saturday	0	0
6	6	Sunday	0	0
7	0	Monday	10	5
8	1	Tuesday	6	7
9	2	Wednesday	10	5
10	3	Thursday	12	4
11	4	Friday	16	2
12	5	Saturday	0	0
13	6	Sunday	0	0
14	0	Monday	10	5
15	1	Tuesday	8	6
16	2	Wednesday	8	6
17	3	Thursday	14	3
18	4	Friday	12	4
19	5	Saturday	0	0
20	6	Sunday	0	0

…

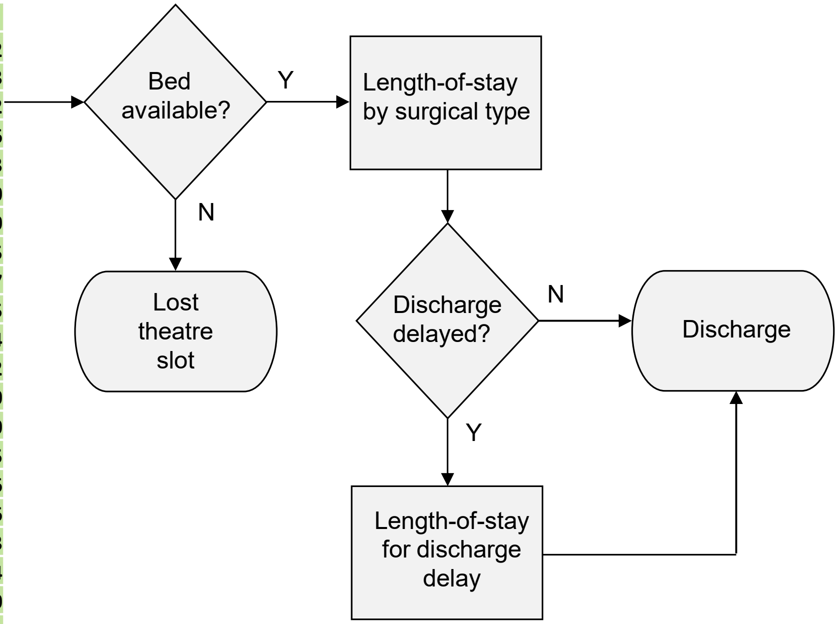

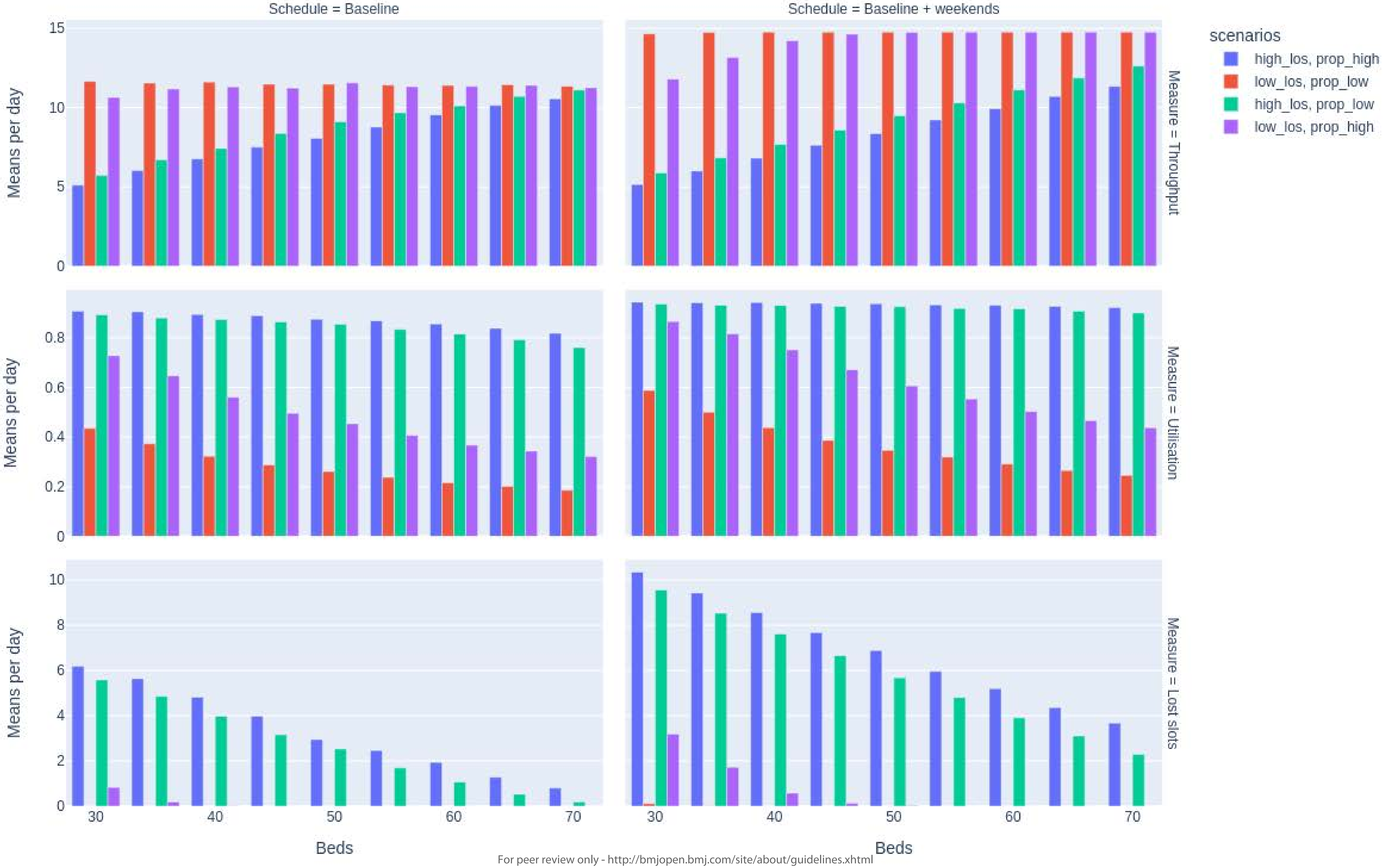
Schedule = Baseline
Schedule = Baseline + weekends
scenarios
high_los, prop_high
low_los, prop_low
high_los, prop_low
low_los, prop_high
Measure = Throughput
Measure = Utilisation
Measure = Lost slots
Means per day
Beds
0
5
10
15
0.2
0.4
0.6
0.8
2
4
6
8
30
40
50
60
70

Supplementary Material 1
STRESS-DES: Strengthening the Reporting of Empirical Simulation Studies
A standardised checklist to improve the reporting of discrete-event simulation models

Monks, T., Currie, C. S., Onggo, B. S., Robinson, S., Kunc, M., & Taylor, S. J. (2019). Strengthening the reporting of empirical simulation studies: Introducing the STRESS guidelines. Journal of Simulation, 13(1), 55-67. https://doi.org/10.1080/17477778.2018.1442155

1.0 Objectives
1.1 Purpose of the model

The HEP orthopaedic capacity planning model provides a tool for healthcare planners, managers, and clinicians to support capacity and efficiency decisions for orthopaedic services. It includes planning for two classes of surgery:

- Primary
- Revision

With five surgical types:

- Primary total hip replacement
- Primary total knee replacement
- Primary unicompartmental knee replacement
- Revision total hip replacement
- Revision total knee replacement

1.2 Model outputs

For each scenario configuration, the model outputs:

(i) Bed utilisation (mean/CI per day/weekday)

(ii) Total mean surgical throughput (by surgical type).

(iii) 'Lost theatre slots' which represent a mismatch between demand (patients scheduled for surgery), and capacity (beds available)

1.3 Experimental aims

A set of 72 indicative experimental parameter configurations were investigated:

Bed numbers between 30 and 70 beds (in increments of 5 beds; a total of 9 bed parameters) were investigated with each of two theatre schedules. The Baseline theatre schedule (4 theatres, 3 sessions per day: (i) revision *or* two primaries; (ii) revision *or* two primaries; (iii) one primary) with 5-day working. The Baseline + weekend schedule uses the same daily theatre allocations, and theatre numbers, with 7-day working. For each of these parameter changes, four scenarios were run for lengths-of-stay (baseline, 25% baseline) and for proportion delayed (baseline, 25% baseline). This totals 72 combinations. Reducing lengths-of-stay and proportion with a discharge delay by 75% brings them in line with national benchmarks. Other scenarios for increasing theatre capacity and changing theatre activity can additionally be investigated in the model.

2.0 Model logic
2.1 Base model overview diagram

For peer review only

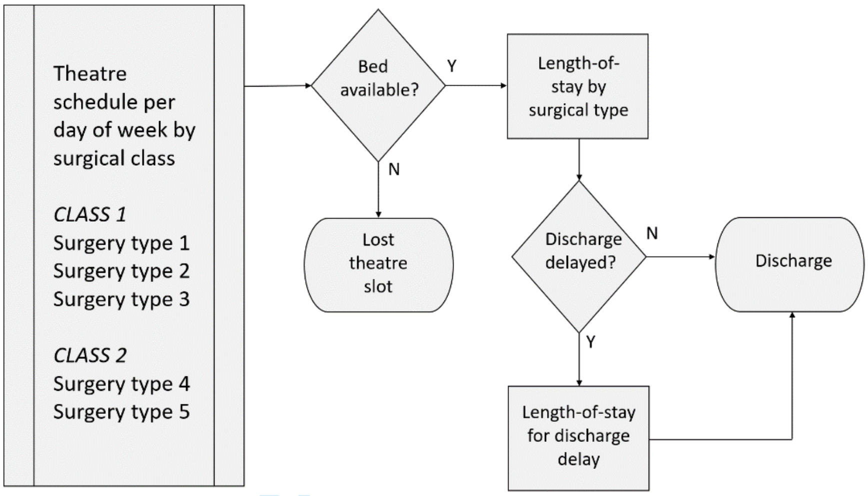

2.2 Base model logic

Patients arrive into the model deterministically using a pre-defined theatre schedule which allocates surgical classes (primary, revision) across a number of sessions per weekday, and a number of theatres per weekday. Surgical types are allocated by probability to each class. Each surgical type samples a length-of-stay from a lognormal distribution fitted to historical data. Using stochastic routing, a proportion of patients will have a delay to their discharge, for example waiting for out-of-hospital care. These patients have an additional length-of-stay sampled from a lognormal distribution. If there is no bed available for a patient scheduled for surgery, this patient represents a 'lost slot'.

2.3 Scenario logic

- Patients arrive into the model according to a pre-defined theatre schedule. The schedule is an experimental parameter. Per weekday, the number of surgery sessions, the allocation of surgical classes per session, and the number of theatres available can be defined.
- Within the model which is made available in streamlit.io, the simulation parameters which can be changed are: number of beds; mean lengths-of-stay per surgical procedure; mean length-of-stay of patients whose discharge is delayed; proportion of patients whose discharge is delayed. Each simulation scenario can be run with the baseline theatre schedule alone, or additionally with a user-defined schedule.
- Within the model notebook, additional parameters can be changed. These include: stdev of all lengths of stay; proportions of surgical types; length-of-stay distributions.

2.4 Components

- A theatre schedule defines deterministic arrivals by patient class
- Patients renege after 0.5-1 day (uniform) if no bed is available ('lost slots')
- Each class has attributes associated with surgical procedure, or delayed discharge
- A single service node
- A single resource: beds
- FIFO queue discipline

3.0 Data

Routinely collected data from North Bristol NHS Trust was used to identify patients receiving elective joint arthroplasty between January 2016 and December 2019.

The Trust's electronic health records (EHR) were used to identify elective joint arthroplasty using a combination of OPCS4 procedure and surgical site codes. Five core elective orthopedic surgical

For peer review only - http://bmjopen.bmj.com/site/about/guidelines.xhtml

procedure types were identified and verified. A small number of short day-case ‘hip resurfacing’ surgeries [n=52] were removed from the dataset. The five remaining surgical types were classified into two classes: (I) Primary (primary hip replacement [p-THR n=3057; 51%], primary knee replacement [p-TKR; n=2302; 38%], uni-compartmental knee replacement [p-UKR; n=679; 11%]); (II) Revision (revision hip replacement [r-THR; n=482; 55%], revision knee replacement [r-TKR; n=392; 45%]). Most patients did not remain in hospital once they were medically fit for discharge, however a proportion of patients in the EHR had a recorded medically fit for discharge date which preceded their actual discharge date (n=529; 7.6%).

A statistical probability distribution was fitted to each type of surgical procedure using the EHR data. The fitted length-of-stay parameters [procedure, mean days (u), standard deviation days (sd)] are: p-THR, u=4.4, sd=2.9 days; p-TKR u=4.7 days, sd=2.8; p-UKR u=2.9 days, sd=2.1; r-THR, u=6.9, sd=7.0; r-TKR, u=7.2, sd=7.6; delayed discharge, u=16.5; sd=15.1).

4.0 Experimentation

4.1 Initialisation

The model uses a warm-up period of 35 days based on visual inspection of outputs. The model is non-terminating.

4.2 Run Length

The model runs for 70 days. This can be changed by the user. Multiple independent replications are undertaken to control confidence intervals. The number of replications is determined using MORE plots, and is set to 50 by default.

Nelson, B. L. (2008) The MORE plot: Displaying measures of risk & error from simulation output. In *2008 Winter Simulation Conference* (pp. 413-416). IEEE.

5.0 Implementation

5.1 Software of programming language

The simulation was developed using Python 3.8. A conda software environment is used to manage versions on a local machine. Software versions are:

- jupyterlab=3.4.6
- jupyterlab-spellchecker=0.7.2
- matplotlib=3.5.3
- numpy=1.23.2
- pandas=1.4.4
- pip=22.2.2
- python=3.8.12
- scipy=1.9.1
- simpy=4.0.1
- streamlit==1.17.0

5.2 Random sampling

All sampling uses `numpy.random.Generator`. Common random number streams are used in the model, created through seed vectors (one seed for each activity in each replication).

5.3 Model execution

Simpy implements a process-based simulation worldview.

5.4 System specification

The model was run on Intel i7-12700H CPU with 32GB RAM running Pop!_OS 22.04 Linux.

For peer review only

6.0 Code access

Code, input parameters and output data used for results in this paper are available on GitHub: AliHarp/HEP: Model of orthopaedic ward (github.com)

They are permanently archived using Zenodo (HEP | Zenodo).

All are licensed using MIT permissive license MIT License | Choose a License.

For peer review only

Supplementary Material 2

Table S1: OPCS-4 codes used to identify hip and knee arthroplasty surgery

PRIMARY TOTAL HIP REPLACEMENT		
4 Character Code	Description	
W37.1	Primary total prosthetic replacement of hip joint using cement	
W37.8	Other specified total prosthetic replacement of hip joint using cement	
W37.9	Unspecified total prosthetic replacement of hip joint using cement	
W38.1	Primary total prosthetic replacement of hip joint not using cement	
W38.8	Other specified total prosthetic replacement of hip joint not using cement	
W38.9	Unspecified total prosthetic replacement of hip joint not using cement	
W39.1	Primary total prosthetic replacement of hip joint NEC	
W39.8	Other specified other total prosthetic replacement of hip joint	
W39.9	Unspecified other total prosthetic replacement of hip joint	
W43.1	Primary total prosthetic replacement of other joint using cement NEC	
W43.8	Other specified total prosthetic replacement of other joint using cement NEC	
W43.9	Unspecified total prosthetic replacement of other joint using cement NEC	
W44.1	Primary total prosthetic replacement of other joint not using cement NEC	
W44.8	Other specified total prosthetic replacement of other joint not using cement NEC	
W44.9	Unspecified total prosthetic replacement of other joint not using cement NEC	
W45.1	Other primary total prosthetic replacement of other joint NEC	
W45.8	Other specified total prosthetic replacement of other joint NEC	
W45.9	Unspecified total prosthetic replacement of other joint NEC	
W52.1	Primary prosthetic replacement of articulation of bone using cement NEC	
W52.8	Other specified prosthetic replacement of articulation of bone using cement NEC	
W52.9	Unspecified prosthetic replacement of articulation of bone using cement NEC	
W53.1	Primary prosthetic replacement of articulation of bone not using cement NEC	
W53.8	Other specified prosthetic replacement of articulation of bone not using cement NEC	

For peer review only

W53.9	Unspecified prosthetic replacement of articulation of bone not using cement NEC	
W54.1	Primary prosthetic replacement of articulation of bone NEC	
W54.8	Other specified prosthetic replacement of articulation of bone NEC	
W54.9	Unspecified prosthetic replacement of articulation of bone NEC	
W93.1	Primary hybrid prosthetic replacement of hip joint using cemented acetabular component	
W93.8	Other specified hybrid prosthetic replacement of hip joint using cemented acetabular component	
W93.9	Unspecified hybrid prosthetic replacement of hip joint using cemented acetabular component	
W94.1	Primary hybrid prosthetic replacement of hip joint using cemented femoral component	
W94.8	Other specified hybrid prosthetic replacement of hip joint using cemented femoral component	
W94.9	Unspecified hybrid prosthetic replacement of hip joint using cemented femoral component	
W95.1	Primary hybrid prosthetic replacement of hip joint using cement NEC	
W95.8	Other specified hybrid prosthetic replacement of hip joint using cement	
W95.9	Unspecified hybrid prosthetic replacement of hip joint using cement	
**RESURFACING / RECONSTRUCTION**		
4 Character Code	Description	
W58.1	Primary resurfacing arthroplasty of joint	
W58.8	Other specified reconstruction of joint	
W58.9	Unspecified other reconstruction of joint	
**LOCATION OF SURGERY**		
4 Character Code	Description	
Z75.6	Acetabulum	
Z76.1	Head of femur	
Z84.3	Hip joint	
**REVISION HIP REPLACEMENT**		
4 Character Code	Description	

For peer review only

W37.0	Conversion from previous cemented total prosthetic replacement of hip joint	
W37.2	Conversion to total prosthetic replacement of hip joint using cement	
W37.3	Revision of total prosthetic replacement of hip joint using cement	
W37.4	Revision of one component of total prosthetic replacement of hip joint using cement	
W38.0	Conversion from previous uncemented total prosthetic replacement of hip joint	
W38.2	Conversion to total prosthetic replacement of hip joint not using cement	
W38.3	Revision of total prosthetic replacement of hip joint not using cement	
W38.4	Revision of one component of total prosthetic replacement of hip joint not using cement	
W39.0	Conversion from previous total prosthetic replacement of hip joint NEC	
W39.2	Conversion to total prosthetic replacement of hip joint NEC	
W39.3	Revision of total prosthetic replacement of hip joint NEC	
W39.5	Revision of one component of total prosthetic replacement of hip joint NEC	
W43.0	Conversion from previous cemented total prosthetic replacement of joint NEC	+ Site code
W43.2	Conversion to total prosthetic replacement of joint using cement NEC	+ Site code
W43.3	Revision of total prosthetic replacement of joint using cement NEC	+ Site code
W43.4	Revision of one component of total prosthetic replacement of joint using cement NEC	+ Site code
W44.0	Conversion from previous uncemented total prosthetic replacement of joint NEC	+ Site code
W44.2	Conversion to total prosthetic replacement of joint not using cement NEC	+ Site code
W44.3	Revision of total prosthetic replacement of joint not using cement NEC	+ Site code
W44.4	Revision of one component of total prosthetic replacement of joint not using cement NEC	+ Site code
W45.0	Conversion from previous total prosthetic replacement of joint NEC	+ Site code
W45.2	Conversion to total prosthetic replacement of joint NEC	+ Site code
W45.3	Revision of total prosthetic replacement of joint NEC	+ Site code
W45.5	Revision of one component of total prosthetic replacement of joint NEC	+ Site code
W46.2	Conversion to prosthetic replacement of head of femur using cement	

For peer review only

W46.3	Revision of prosthetic replacement of head of femur using cement	
W47.2	Conversion to prosthetic replacement of head of femur not using cement	
W47.3	Revision of prosthetic replacement of head of femur not using cement	
W48.2	Conversion to prosthetic replacement of head of femur NEC	
W48.3	Revision of prosthetic replacement of head of femur NEC	
W52.0	Conversion from previous cemented prosthetic replacement of articulation of bone NEC	+ Site code
W52.2	Conversion to prosthetic replacement of articulation of bone using cement NEC	+ Site code
W52.3	Revision of prosthetic replacement of articulation of bone using cement NEC	+ Site code
W53.0	Conversion from previous uncemented prosthetic replacement of articulation of bone NEC	+ Site code
W53.2	Conversion to prosthetic replacement of articulation of bone not using cement NEC	+ Site code
W53.3	Revision of prosthetic replacement of articulation of bone not using cement NEC	+ Site code
W54.0	Conversion from previous prosthetic replacement of articulation of bone NEC	+ Site code
W54.2	Conversion to prosthetic replacement of articulation of bone NEC	+ Site code
W54.3	Revision of prosthetic replacement of articulation of bone NEC	+ Site code
W57.4	Conversion to excision arthroplasty of joint	+ Site code
W58.0	Conversion from previous resurfacing arthroplasty of joint	+ Site code
W58.2	Revision of resurfacing arthroplasty of joint	+ Site code
W93.0	Conversion from previous hybrid prosthetic replacement of hip joint using cemented acetabular component	
W93.2	Conversion to hybrid prosthetic replacement of hip joint using cemented acetabular component	
W93.3	Revision of hybrid prosthetic replacement of hip joint using cemented acetabular component	
W94.0	Conversion from previous hybrid prosthetic replacement of hip joint using cemented femoral component	
W94.2	Conversion to hybrid prosthetic replacement of hip joint using cemented femoral component	
W94.3	Revision of hybrid prosthetic replacement of hip joint using cemented femoral component	
W95.0	Conversion from previous hybrid prosthetic replacement of hip joint using cement NEC	
W95.2	Conversion to hybrid prosthetic replacement of hip joint using cement NEC	
W95.3	Revision of hybrid prosthetic replacement of hip joint using cement NEC	

For peer review only

**PRIMARY TOTAL KNEE REPLACEMENT**		
4 Character Code	Description	
W40.1	Primary total prosthetic replacement of knee joint using cement	
W40.8	Other specified total prosthetic replacement of knee joint using cement	
W40.9	Unspecified total prosthetic replacement of knee joint using cement	
W41.1	Primary total prosthetic replacement of knee joint not using cement	
W41.8	Other specified total prosthetic replacement of knee joint not using cement	
W41.9	Unspecified total prosthetic replacement of knee joint not using cement	
W42.1	Primary total prosthetic replacement of knee joint NEC	
W42.8	Other specified other total prosthetic replacement of knee joint	
W42.9	Unspecified other total prosthetic replacement of knee joint	
O18.1	Primary hybrid prosthetic replacement of knee joint using cement	
O18.8	Other specified hybrid prosthetic replacement of knee joint using cement	
O18.9	Unspecified hybrid prosthetic replacement of knee joint using cement	
**PRIMARY UNICONDYLAR / UNICOMPARTMENTAL KNEE OPERATIONS**		
4 Character Code	Description	
W52.1	Primary prosthetic replacement of articulation of bone using cement NEC	Require combination with site + combination codes to ID
W52.8	Other specified prosthetic replacement of articulation of other bone using cement	Require combination with site + combination codes to ID

W52.9	Unspecified prosthetic replacement of articulation of other bone using cement	Require combination with site + combination codes to ID
W53.1	Primary prosthetic replacement of articulation of bone not using cement NEC	Require combination with site + combination codes to ID
W53.9	Unspecified prosthetic replacement of articulation of other bone not using cement	Require combination with site + combination codes to ID
W54.0	Conversion from previous prosthetic replacement of articulation of bone NEC	Require combination with site + combination codes to ID
W54.1	Primary prosthetic replacement of articulation of bone NEC	Require combination with site + combination codes to ID
W54.8	Other specified other prosthetic replacement of articulation of other bone	Require combination with site + combination codes to ID
W54.9	Unspecified other prosthetic replacement of articulation of other bone	Require combination with site + combination codes to ID
W58.1	Primary resurfacing arthroplasty of joint	Require combination with site + combination codes to ID
**SITE OF SURGERY**		
Z76.5	Lower end of femur NEC	
Z77.4	Upper end of tibia NEC	
Z78.7	Patella	Will need care in extracting as PFJ replacement will be coded with this
Z84.4	Patellofemoral joint	Will need care in extracting as PFJ replacement will be coded with this
Z84.5	Tibiofemoral joint	
Z84.6	Knee joint	
**REVISION KNEE REPLACEMENT**		
4 Character Code	Description	
W40.0	Conversion from previous cemented total prosthetic replacement of knee joint	

For peer review only

W40.2	Conversion to total prosthetic replacement of knee joint using cement	
W40.3	Revision of total prosthetic replacement of knee joint using cement	
W40.4	Revision of one component of total prosthetic replacement of knee joint using cement	
W41.0	Conversion from previous uncemented total prosthetic replacement of knee joint	
W41.2	Conversion to total prosthetic replacement of knee joint not using cement	
W41.3	Revision of total prosthetic replacement of knee joint not using cement	
W41.4	Revision of one component of total prosthetic replacement of knee joint not using cement	
W42.0	Conversion from previous total prosthetic replacement of knee joint NEC	
W42.2	Conversion to total prosthetic replacement of knee joint NEC	
W42.3	Revision of total prosthetic replacement of knee joint NEC	
W42.4	Attention to total prosthetic replacement of knee joint NEC	Plus Y03.2 (Renewal of prosthesis in organ NOC) or Y03.7 (Removal of prosthesis from organ NOC)
W42.5	Revision of one component of total prosthetic replacement of knee joint NEC	
W42.6	Arthrolysis of total prosthetic replacement of knee joint	
W58.0	Conversion from previous resurfacing arthroplasty of joint	
W58.2	Revision of resurfacing arthroplasty of joint	Require combination with site + combination codes to ID
O18.0	Conversion from previous hybrid prosthetic replacement of knee joint using cement	
O18.2	Conversion to hybrid prosthetic replacement of knee joint using cement	
O18.3	Revision of hybrid prosthetic replacement of knee joint using cement	
O18.4	Attention to hybrid prosthetic replacement of knee joint using cement	
W52.0	Conversion from previous cemented prosthetic replacement of articulation of bone NEC	Require combination with site + combination codes to ID
W52.2	Conversion to prosthetic replacement of articulation of bone using cement NEC	Require combination with site + combination codes to ID

For peer review only

W52.3	Revision of prosthetic replacement of articulation of bone using cement NEC	Require combination with site + combination codes to ID
W53.0	Conversion from previous uncemented prosthetic replacement of articulation of bone NEC	Require combination with site + combination codes to ID
W53.2	Conversion to prosthetic replacement of articulation of bone not using cement NEC	Require combination with site + combination codes to ID
W53.3	Revision of prosthetic replacement of articulation of bone not using cement NEC	Require combination with site + combination codes to ID
W54.0	Conversion from previous prosthetic replacement of articulation of bone NEC	Require combination with site + combination codes to ID
W54.2	Conversion to prosthetic replacement of articulation of bone NEC	Require combination with site + combination codes to ID
W54.3	Revision of prosthetic replacement of articulation of bone NEC	Require combination with site + combination codes to ID
W54.4	Attention to prosthetic replacement of articulation of bone NEC	Require combination with site + combination codes to ID
W55.3	Conversion to prosthetic interposition arthroplasty of joint	Require combination with site + combination codes to ID
W56.4	Conversion to interposition arthroplasty of joint NEC	Require combination with site + combination codes to ID
W57.4	Conversion to excision arthroplasty of joint	Require combination with site + combination codes to ID
W60.3	Conversion to arthrodesis and extra-articular bone graft NEC	Require combination with site + combination codes to ID
W61.3	Conversion to arthrodesis and articular bone graft NEC	Require combination with site + combination codes to ID
W64.1	Conversion to arthrodesis and internal fixation NEC	Require combination with site + combination codes to ID
W64.2	Conversion to arthrodesis and external fixation NEC	Require combination with site + combination codes to ID

For peer review only
